# Supplementary material for: Metal chloride perovskite thin film based interfacial layer for shielding lithium metal from liquid electrolyte
Source: Nat Commun. 2020 Apr 9;11:1761. doi: 10.1038/s41467-020-15643-9 (PMC7145840; doi:10.1038/s41467-020-15643-9)
Supplement: Supplementary file 1 — Supplementary Information [file 41467_2020_15643_MOESM1_ESM.pdf]

Supplementary Information

Metal chloride perovskite thin film based interfacial layer for shielding lithium metal from liquid electrolyte

Yin et al.

Supplementary Figures

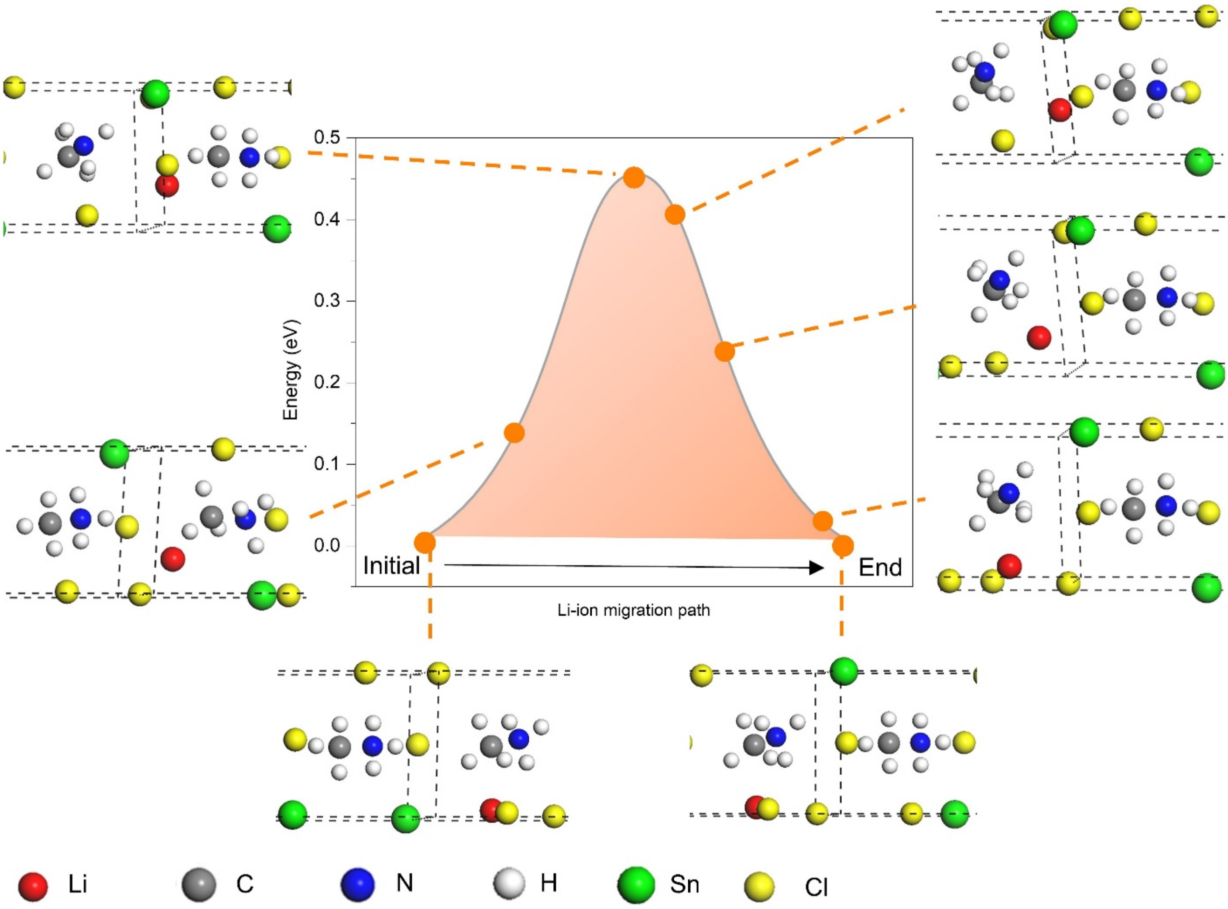

Supplementary Fig. 1 | The calculated potential energy surface value for Li^+ ion's migration and corresponding specific positions of Li^+ ion. The orientation of methylammonium ion (MA^+) in the end state is spontaneously adjusted to the same as the initial state, which is important for the periodicity formation of the Li^+ ion transport pathways.

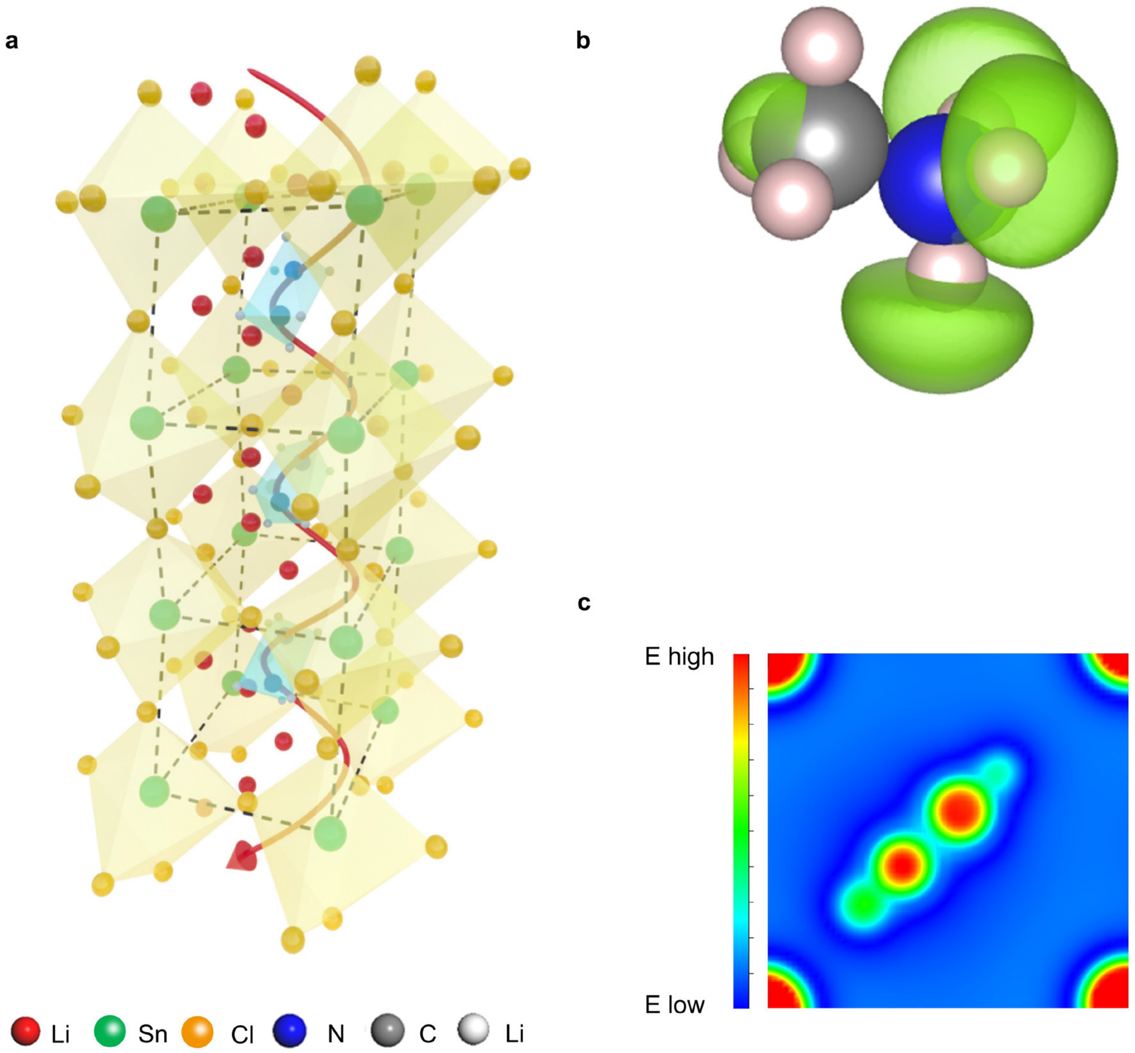

Supplementary Fig. 2 | Calculations of $MASnCl_3$ lattice. a, Schematic of Li^+ ion's migration pathway through the lattice of perovskite. **b,** Charge distribution of MA ion (isosurface level=0.003).**c,** electropotential distribution of (001) crystal face of $MASnCl_3$ lattice.

a

Solute	Concentration	Solvent
$MASnCl_3$	0.5 M	DMF
$MAPbCl_3$	1 M	DMSO

b

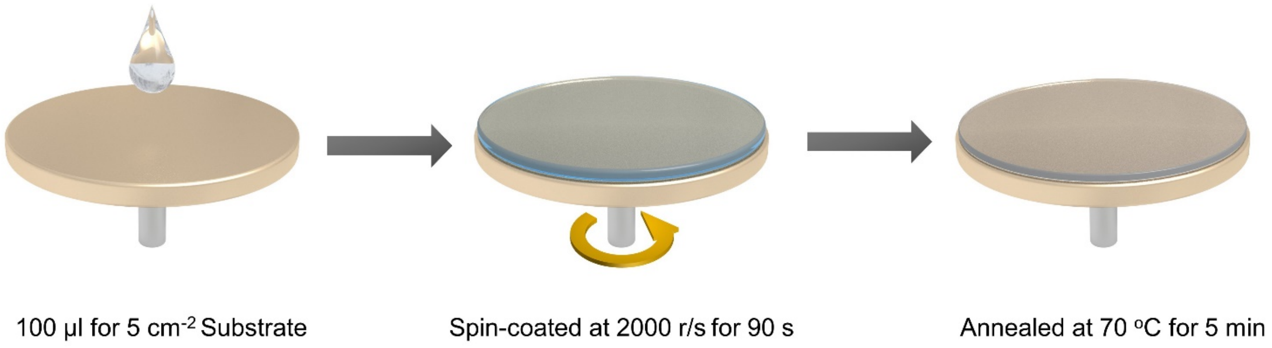

Supplementary Fig. 3 | Preparation of highly-oriented perovskite films. a, Compositions of the $MASnCl_3$ and $MAPbCl_3$ solutions for spin-coating, **b,** Scheme of fabrication procedures, including spin-coating and annealing of $MASnCl_3$ and $MAPbCl_3$ thin films.

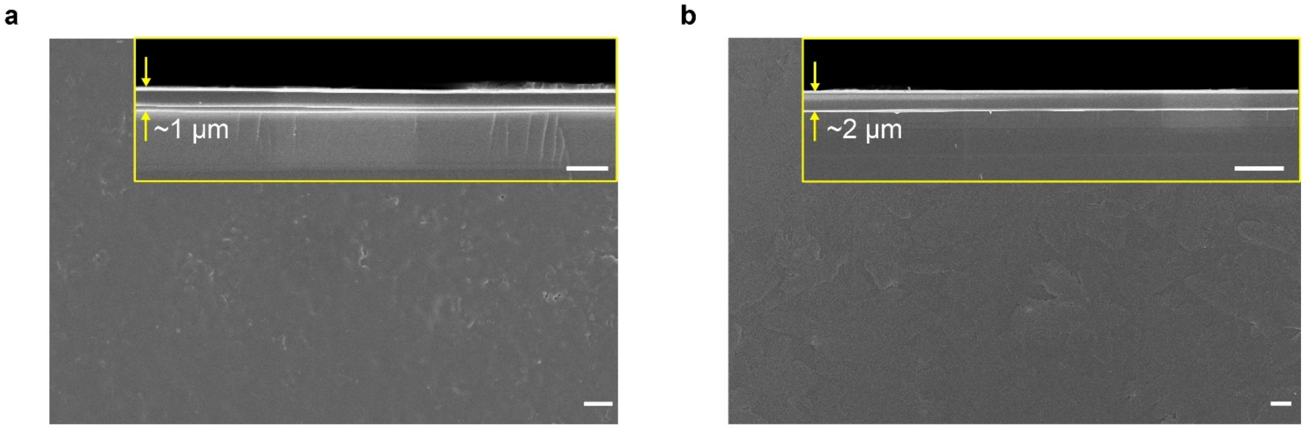

Supplementary Fig. 4 | SEM images showing the surface and cross-sectional morphologies of spin-coated perovskite. a, SEM images showing the surface and cross-sectional morphologies (inset) of spin-coated $MASnCl_3$, **b,** SEM images showing the surface and cross-sectional morphologies (inset) of spin-coated $MAPbCl_3$. Scale bars: 2 μm in a, 5 μm in b.

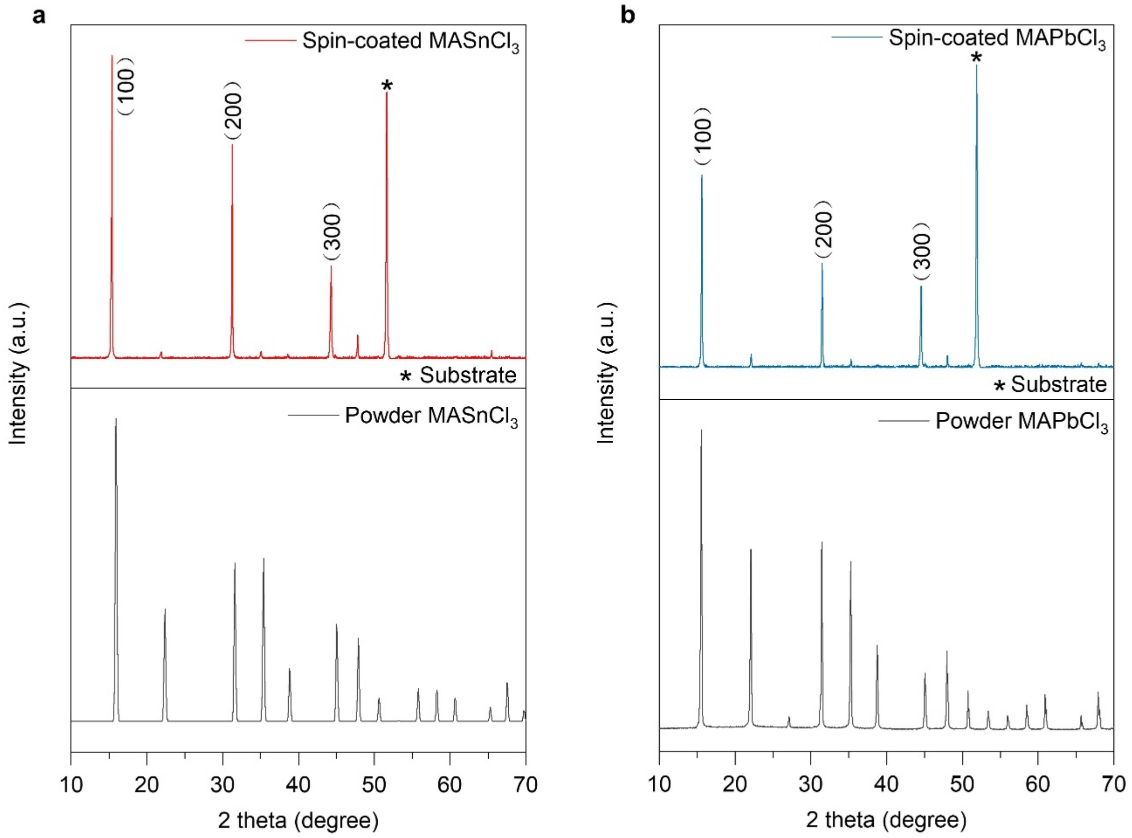

Supplementary Fig. 5 | XRD patterns of spin-coated or powder pervoskite. a, XRD patterns of spin-coated $MASnCl_3$ film and $MASnCl_3$ powder, **b,** XRD patterns of spin-coated $MAPbCl_3$ film and $MAPbCl_3$ powder. Powder XRD patterns are available from previous literatures[1,2].

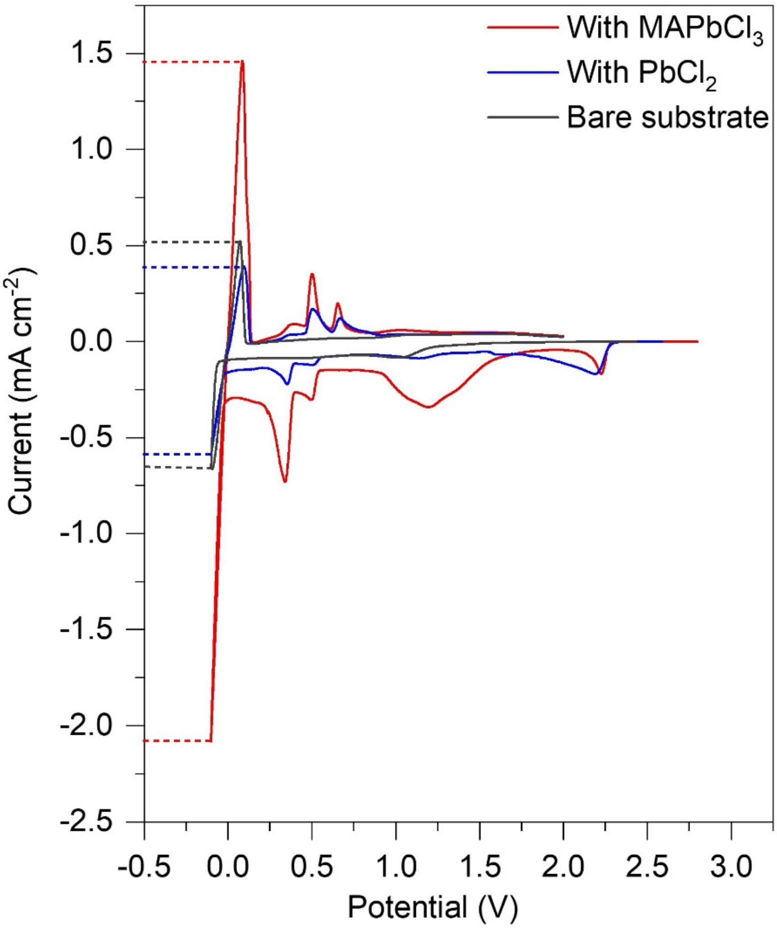

Supplementary Fig. 6 | Cyclic voltammetry curves of cells using Li versus bare, $PbCl_2$-coated or $MAPbCl_3$-coated steel substrates to further demonstrate the advantages of perovskite structure to facilitate Li plating/stripping.

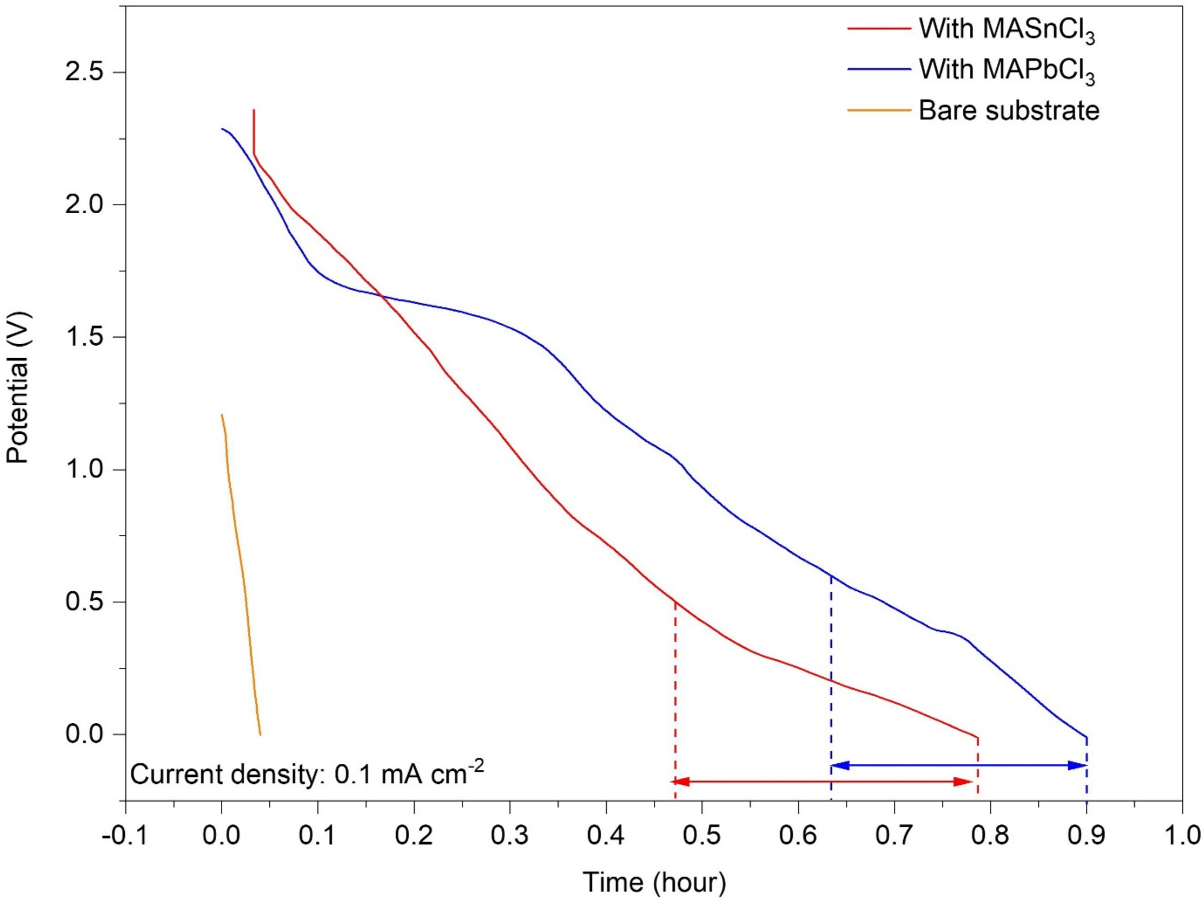

Supplementary Fig. 7 | Galvanostatic deposition curve of bare steel substrate, $MASnCl_3$-coated steel substrate and $MAPbCl_3$-coated steel substrate (versus Li).

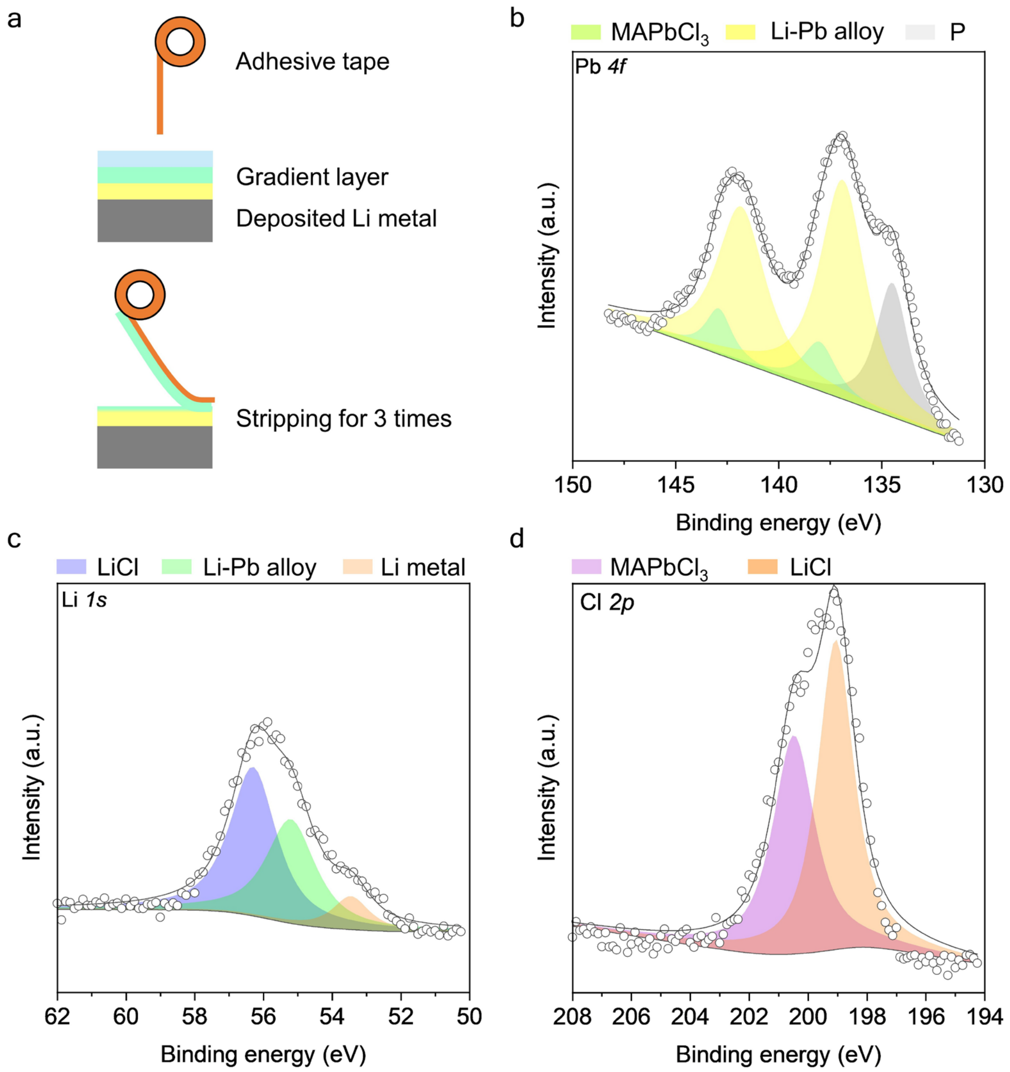

Supplementary Fig. 8 | Depth XPS analysis of the Li-Pb alloy layer formed during the conversion-type electrochemical reaction. a, schematic of the stripping operation via adhesive tape. **b-d,** XPS spectra of Pb 4f **(b)**, Li 1s **(c)** and Cl 2p **(d)** after stripping out the top part of the perovskite thin film.

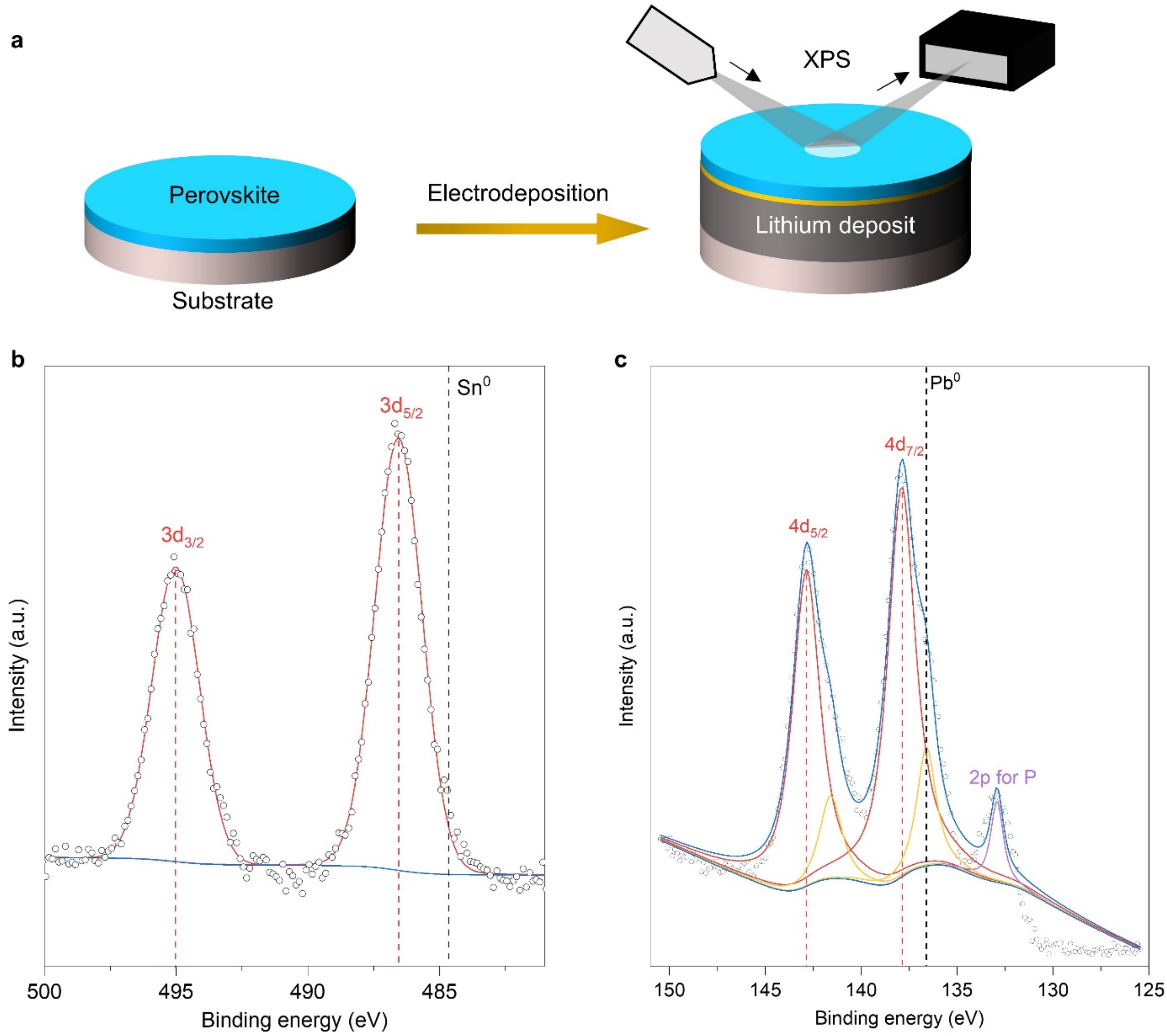

Supplementary Fig. 9 | XPS detection methods and results of perovskite-coated substrates after lithium electrochemical deposition. a, schematic of the XPS detection methods, **b-c,** XPS spectra of Sn on $MASnCl_3$-coated substrate (**b**) and Pb on $MAPbCl_3$-coated substrate (**c**) after electrodeposition of lithium under 2 mA cm^{-2} for 10 h. The samples were thoroughly washed with EC/DEC/DMC mixed solution after electrodeposition and the transfer of the sample was carried out in an argon-filled transfer vessel.

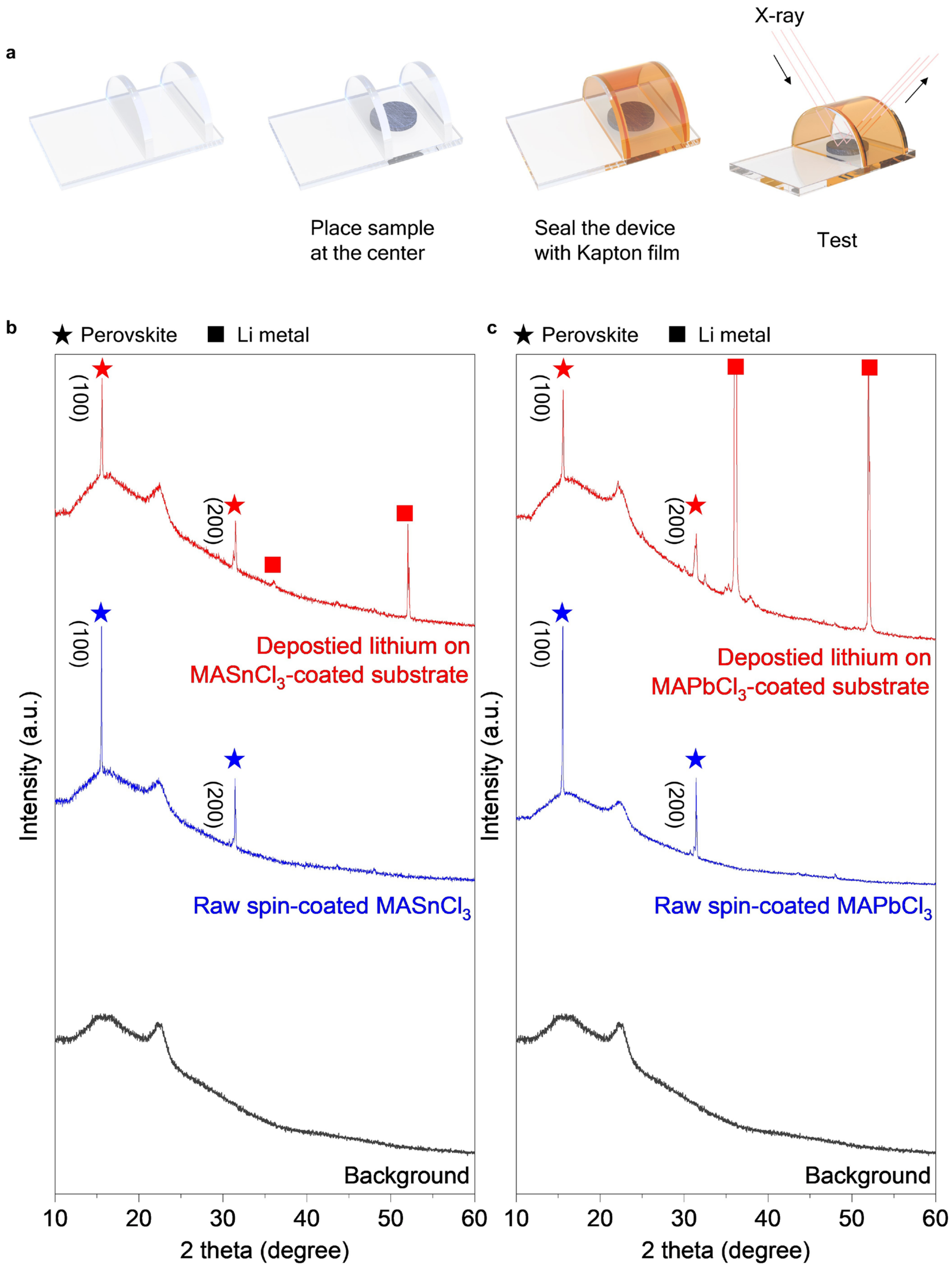

Supplementary Fig. 10 | XRD patterns of deposited Li on perovskite-coated substrate. a, Sealing device and operation procedure for XRD testing. **b,** XRD patterns of deposited Li metal on $MASnCl_3$-coated substrate (red), raw spin-coated $MASnCl_3$ and background (black, generated by sealing device). **c,** XRD patterns of deposited Li metal on $MAPbCl_3$-coated substrate (red), raw spin-coated $MAPbCl_3$ and background (black, generated by sealing device).

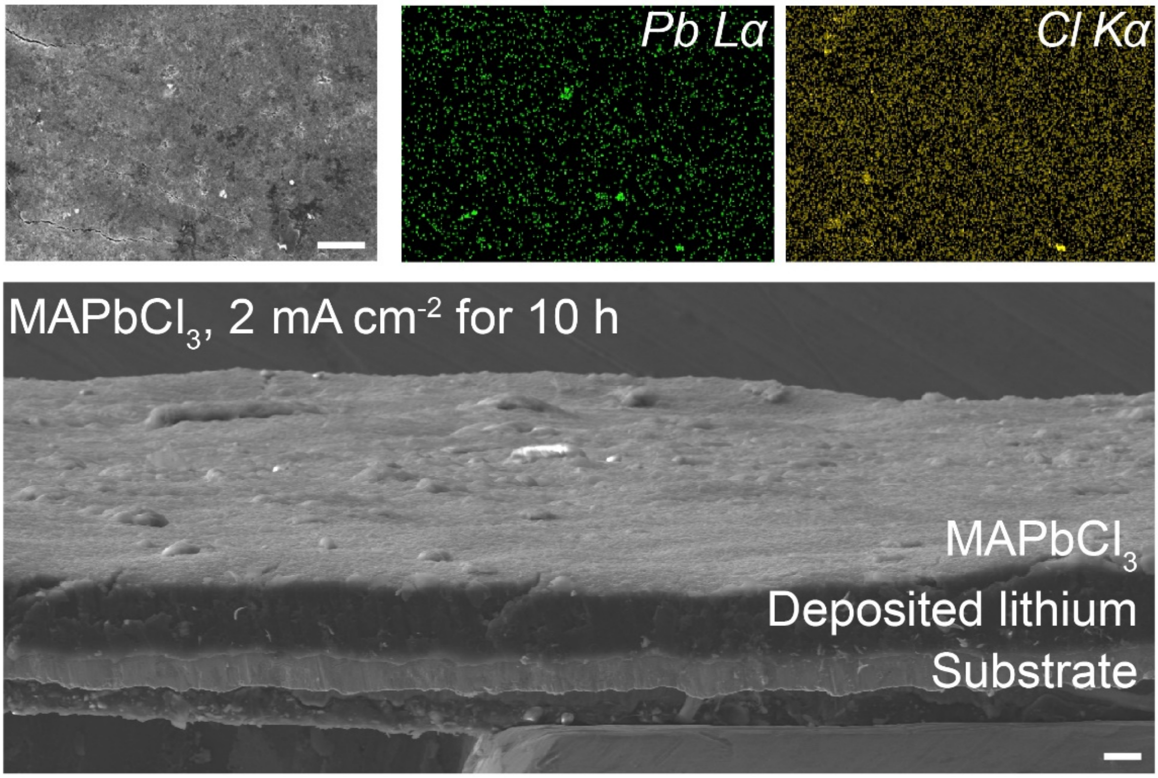

Supplementary Fig. 11 | Morphologies of Li electrodeposited on $MAPbCl_3$-coated substrates and the SEM image with corresponding EDX mappings indicating the distribution of Pb, Cl on the top surface.

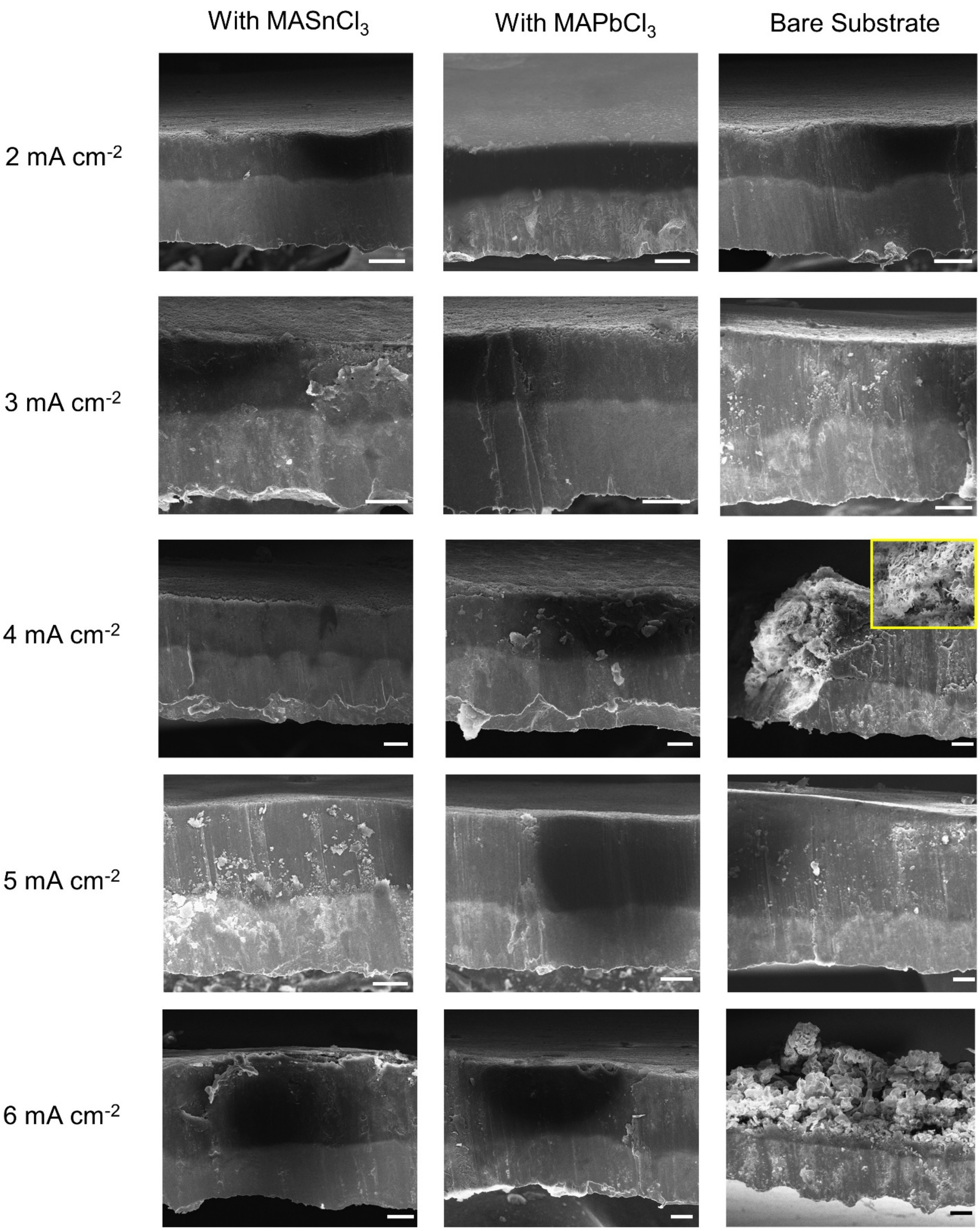

Supplementary Fig. 12 | Cross-sectional morphologies of the Li deposit on $MASnCl_3$-coated substrate, $MAPbCl_3$-coated substrate and bare substrate under different current densities of 2 ~ 6 mA cm^{-2} for 5 hours. Scale bars: 50 µm.

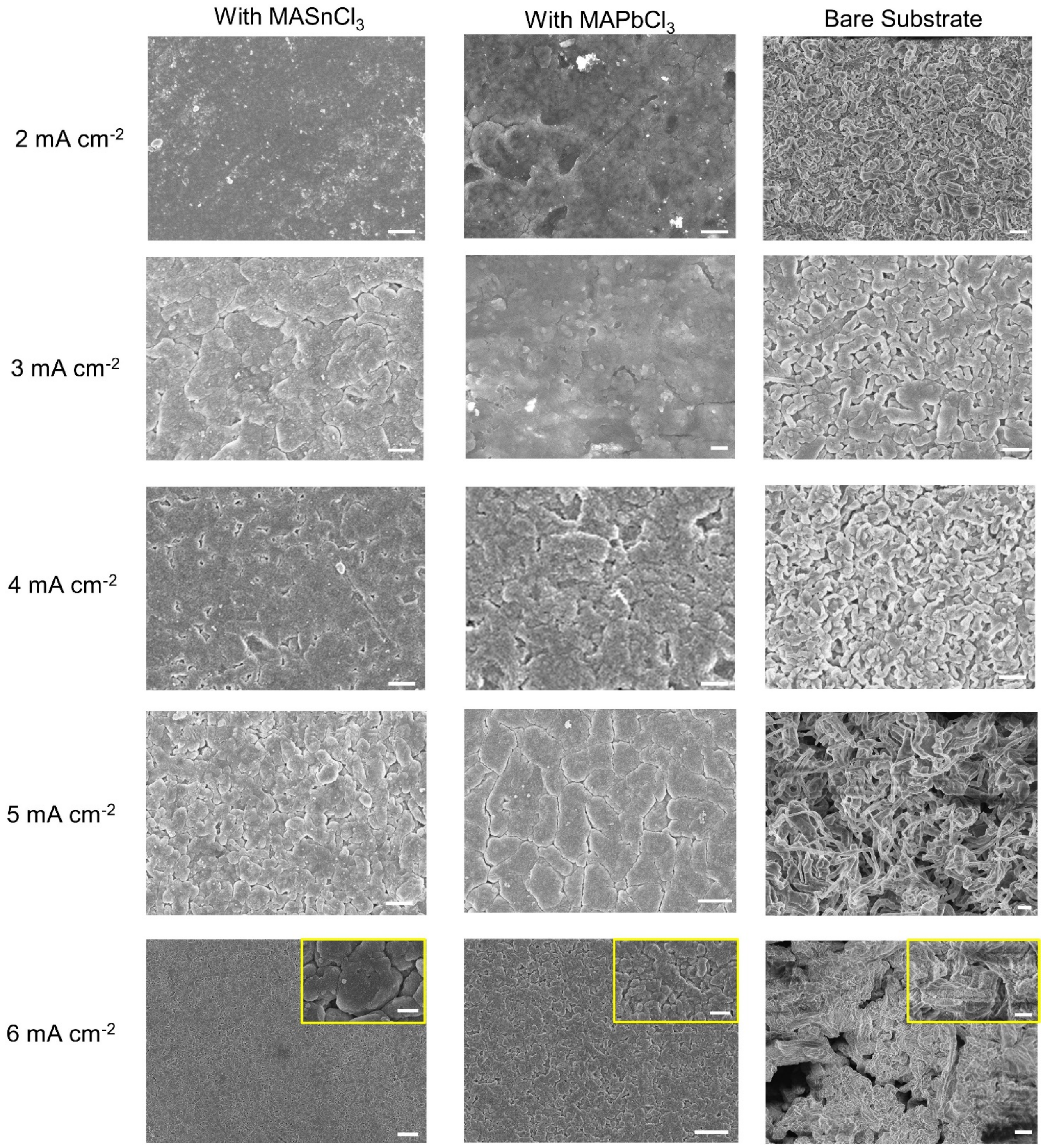

Supplementary Fig. 13 | Surface morphologies of the Li deposit onto $MASnCl_3$-coated substrate, $MAPbCl_3$-coated substrate and bare substrate under different current densities of 2 ~ 6 mA cm^{-2} for 5 hours. Scale bars: 2 μm for all images of current density of 2~5 mA cm^{-2}, 10 μm for large-scale view images for all images of current density of 6 mA cm^{-2}. For yellow-framed insets figures, scale bars are 200 nm, 2 μm and 2 μm, respectively.

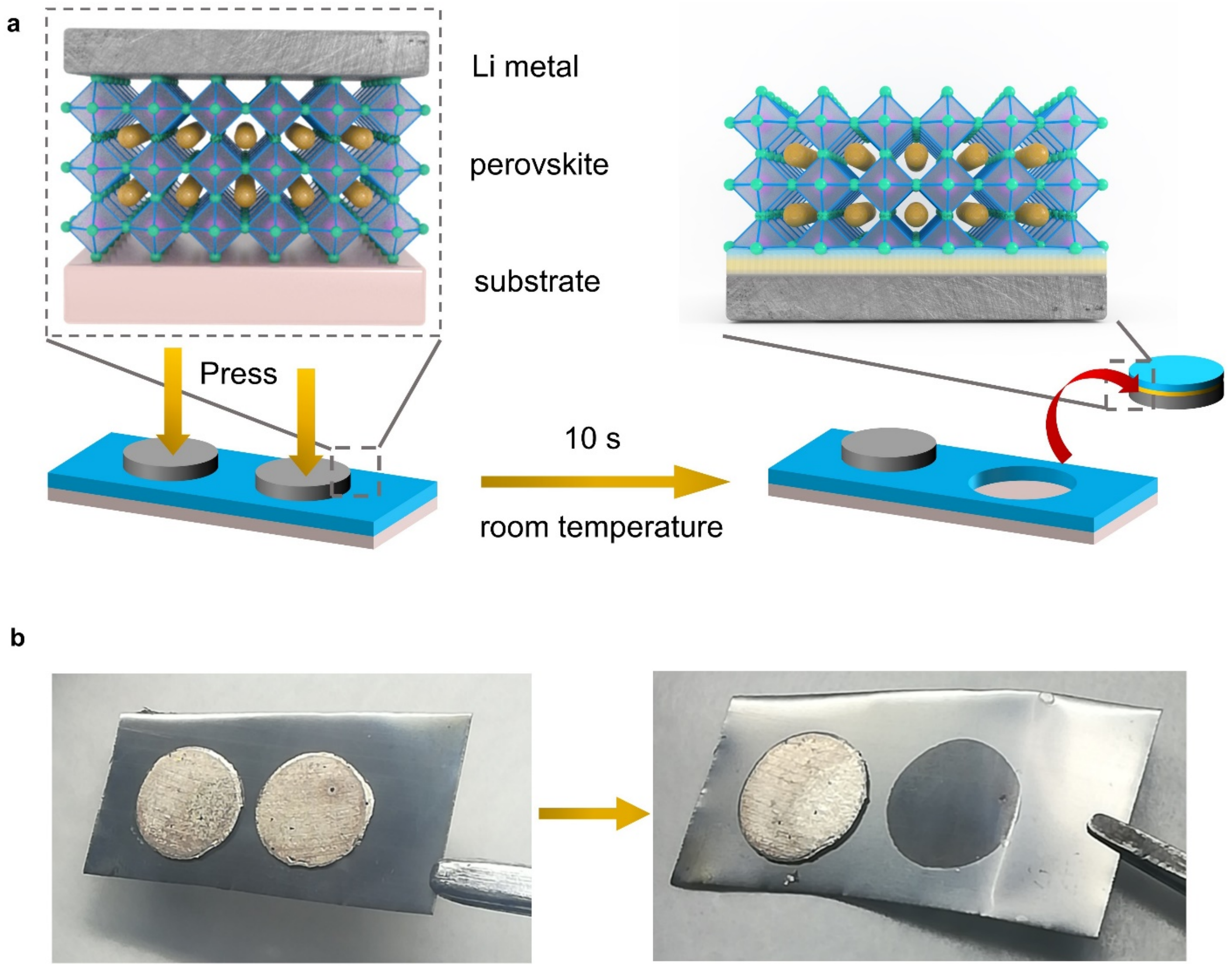

Supplementary Fig. 14 | The solid-state transfer process. a, Illustration of the solid-state transfer process of perovskite film onto lithium surface to form MSC-Li or MPC-Li anodes. **b,** Photos of the perovskite-coated stainless substrate before and after transfer process, which reveals that the perovskite film is well separated from the original substrate.

a

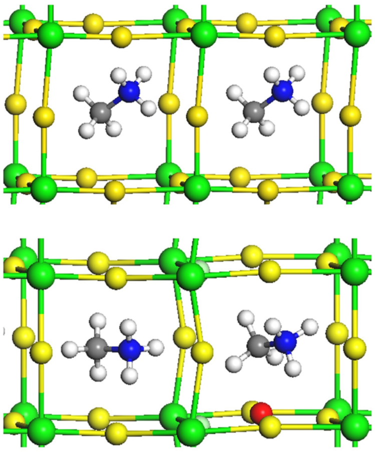

b

Lattice Parameters		
$MASnCl_3$	a	11.36
	b	5.67
	c	5.66
	$\alpha \times \beta \times \gamma$	89 x 89 x 88
Li ions into $MASnCl_3$	a	10.88
	b	5.42
	c	5.36
	$\alpha \times \beta \times \gamma$	88 x 78 x 88

c

Structure	Energy (eV)
Li/$MASnCl_3$	-116.71
$MASnCl_3$	-110.61
Li	-1.217
ΔE (Li intercalation)	-4.88

*ΔE(Li intercalation) = E(Li/$MASnCl_3$)-E($MASnCl_3$)-E(Li)

Supplementary Fig. 15 | Structure and energy calculation of the pristine and lithiated metal chloride perovskite. a, Structure of $MASnCl_3$ lattice (top) and $MASnCl_3$-Li lattice (bottom). **b**, the table of the lattice parameters of $MASnCl_3$ and $MASnCl_3$-Li. **c**, the table of energy calculation of the Li^+ ion intercalation behavior.

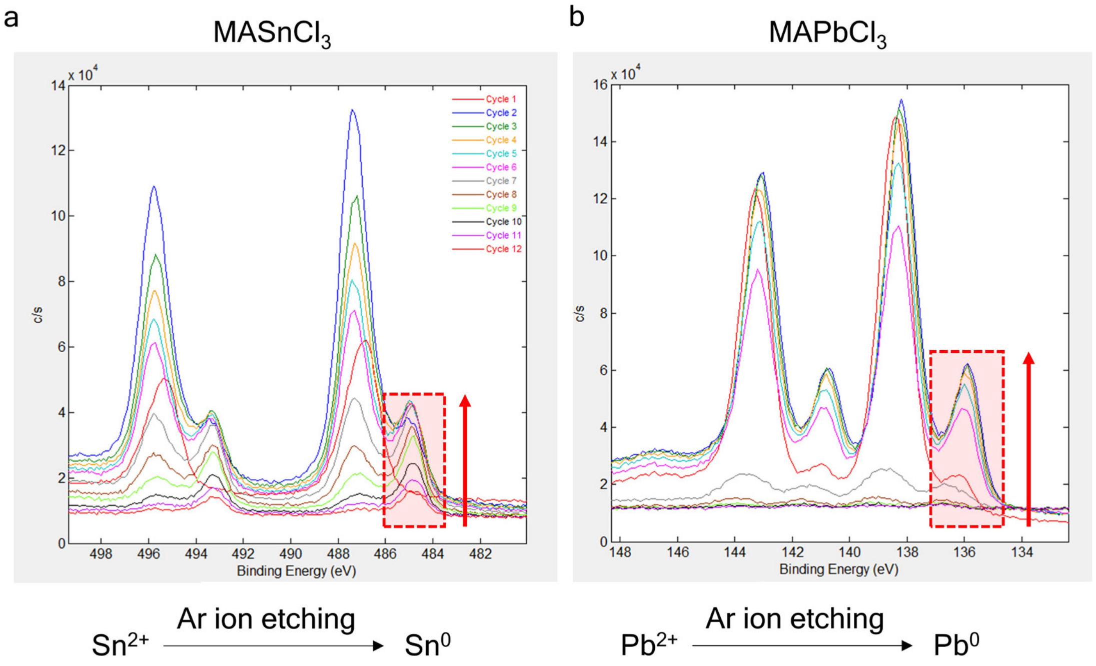

Supplementary Fig. 16 | Damage of perovskite and reduction of metallic elements in perovskite caused by Ar ion sputtering. a, reduction of Sn in $MASnCl_3$ caused by Ar ion sputtering, **b,** reduction of Pb in $MAPbCl_3$ caused by Ar ion sputtering.

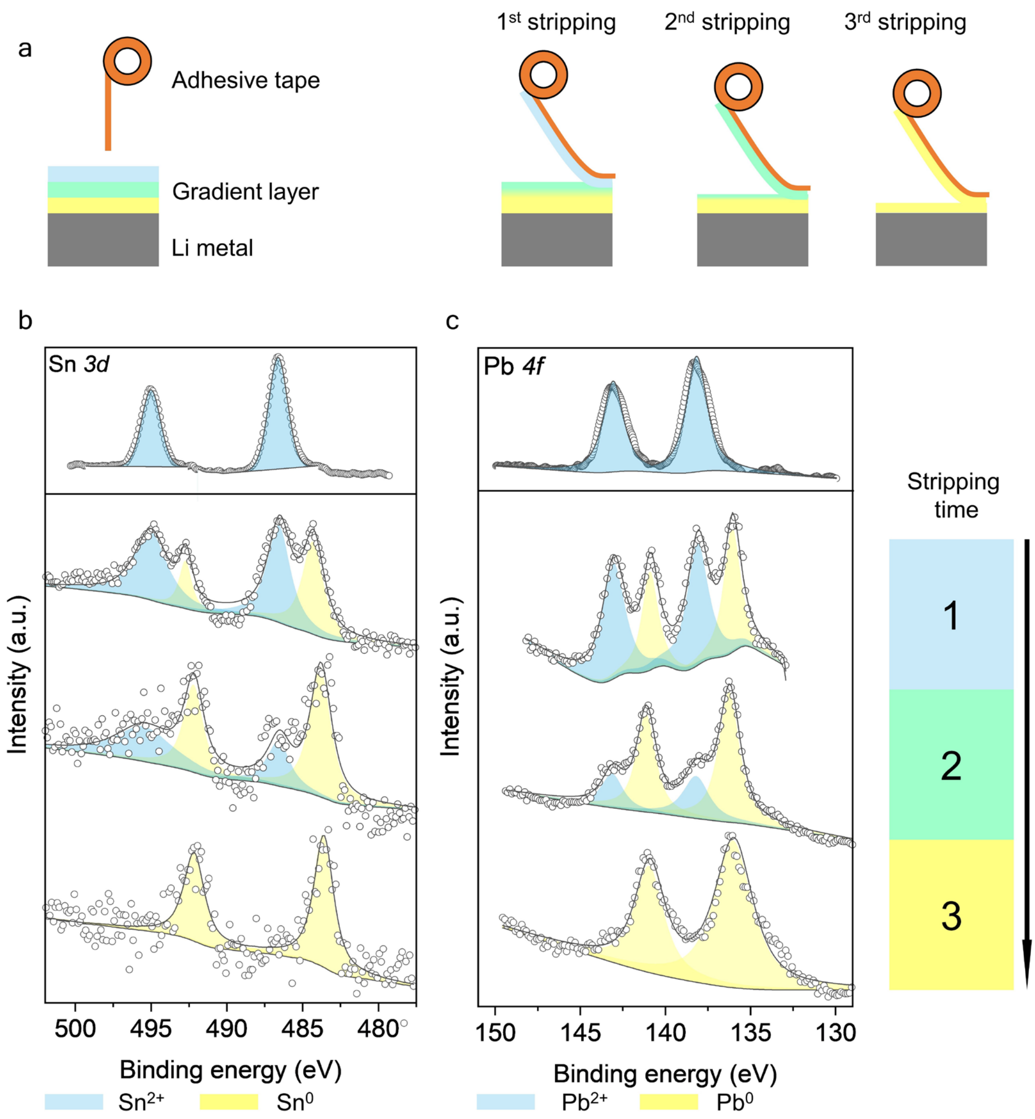

Supplementary Fig. 17 | Depth XPS analysis of the perovskite thin films on the Li metal anodes to show the gradient composition change along the cross-section. a, schematic of the stripping operation via adhesive tape. **b-c,** XPS spectra comparison of Sn 3d **(b)**, Pb 4f **(c)** at different depth exposed by different times of the adhesive tape stripping (the top part data taken from Fig. 3d and Supplementary Fig. 28 as the reference for the comparison).

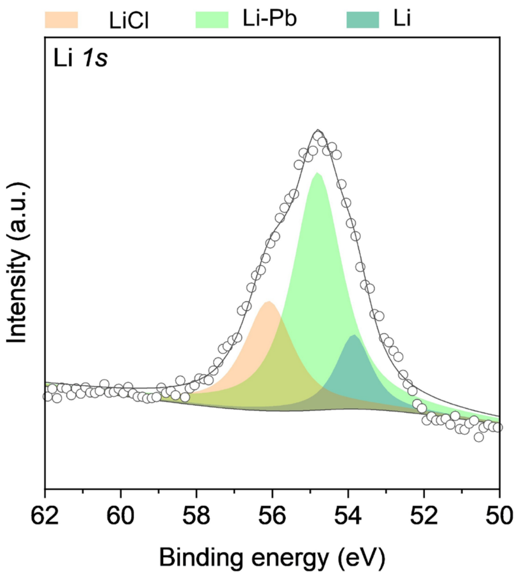

Supplementary Fig. 18 | Li 1s XPS spectrum of MPC-Li after stripping for 3 times.

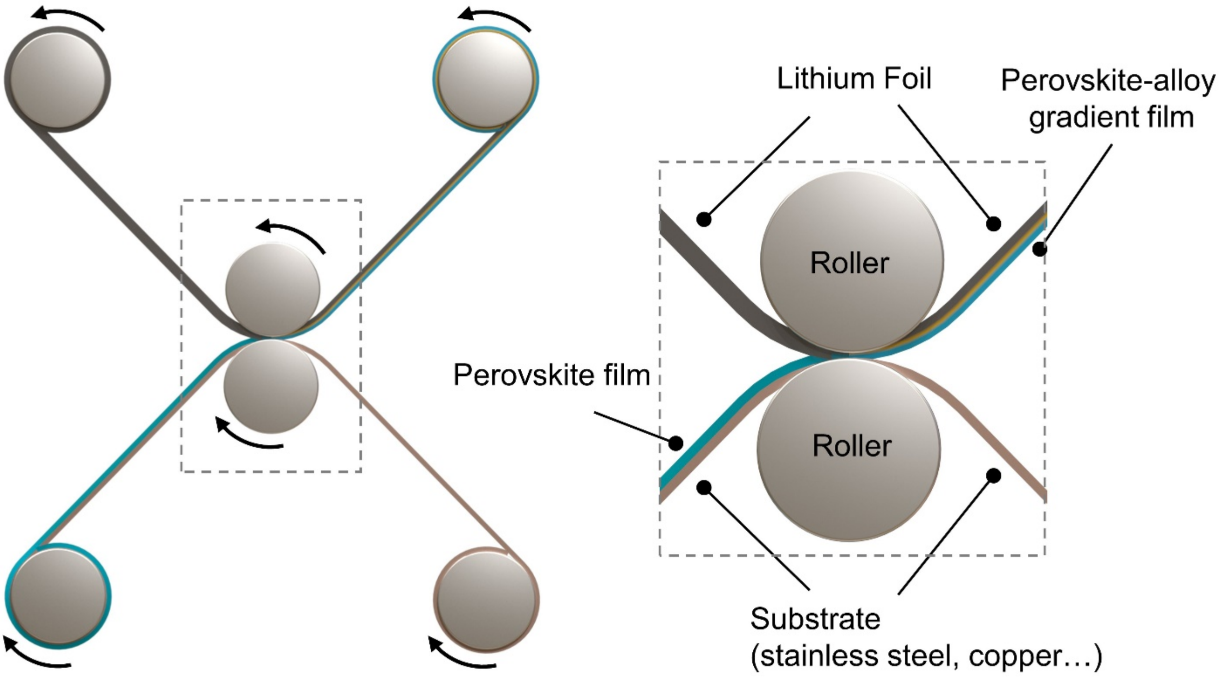

Supplementary Fig. 19 | The schematic illustration of the potential of the solid-state transfer process to be practically applied in the battery industry.

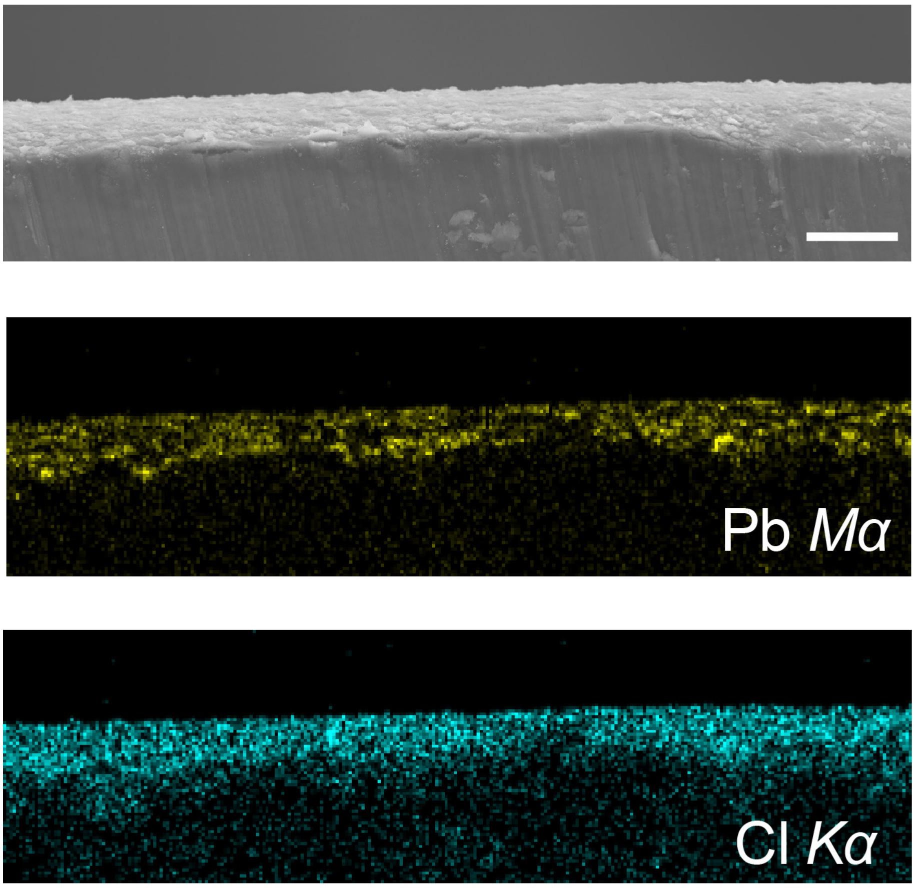

Supplementary Fig. 20 | The schematic illustration of the potential of the solid-state transfer process to be practically applied in the battery industry.

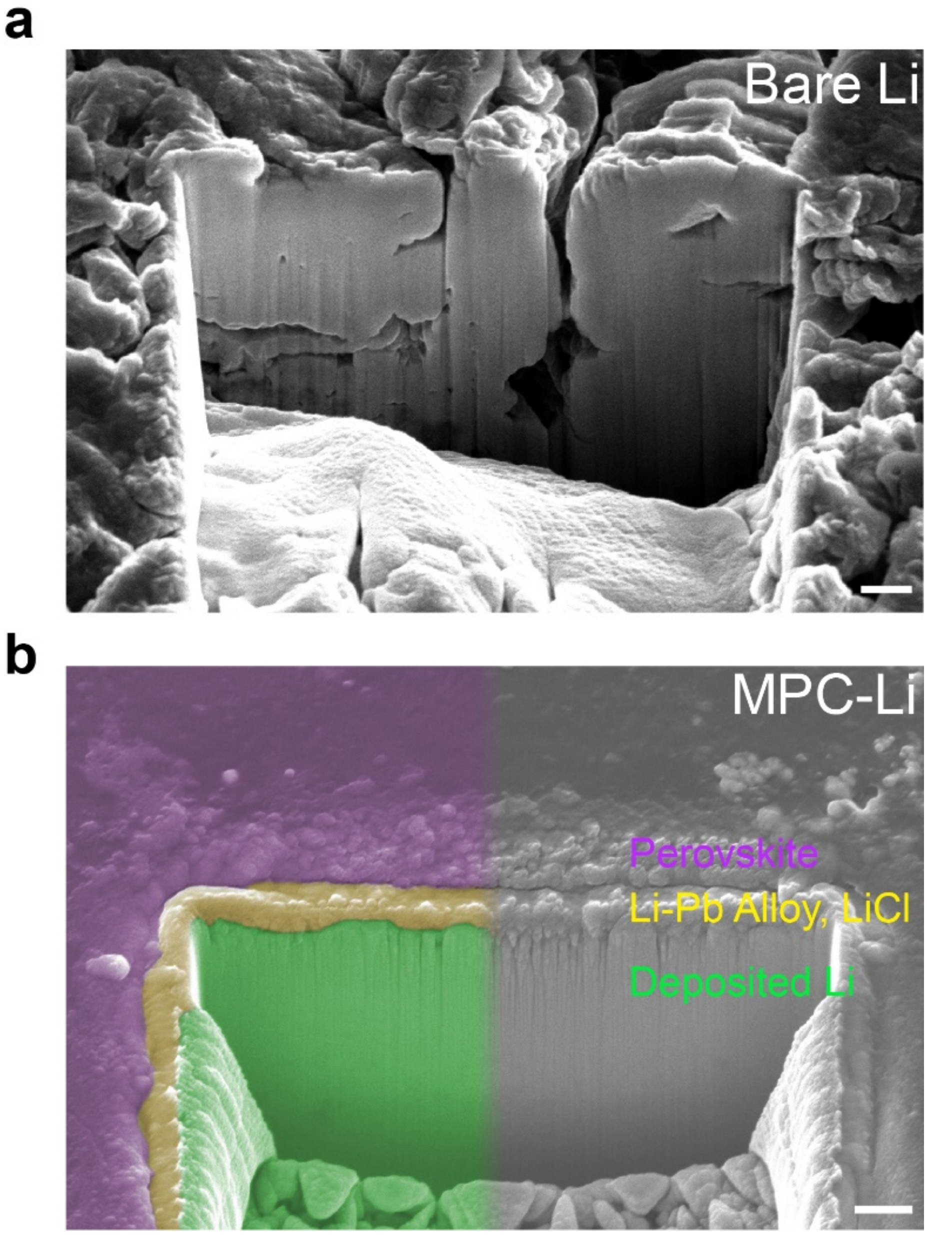

Supplementary Fig. 21 | FIB-processed deep cross-sectional morphologies of bare or perovskite-coated lithium metal electrodes after electrochemical deposition of lithium. a, deep cross-sectional morphology of bare Li after electrodeposition under 4 mA cm^{-2} for 2 h, **b,** deep cross-sectional morphology of MPC-Li after electrodeposition under 4 mA cm^{-2} for 2 h. The different colors based on the contrast difference implies the retention of perovskite-alloy-lithium (purple-yellow-green) gradient structure of the perovskite composite Li anodes. Scale bars: 1 μm.

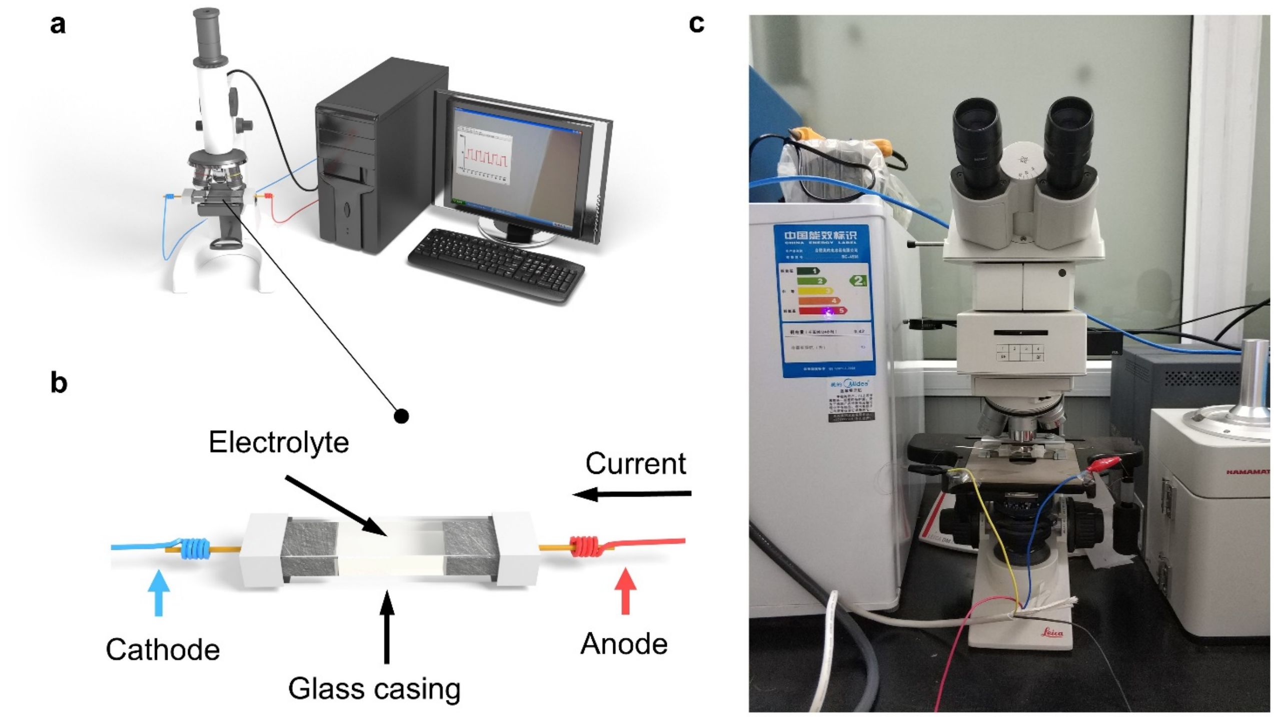

Supplementary Fig. 22 | Devices for visual battery testing. a, Schematic figure of the devices for visual battery testing. **b,** The structure of visual cell. **c,** The photo of the equipment.

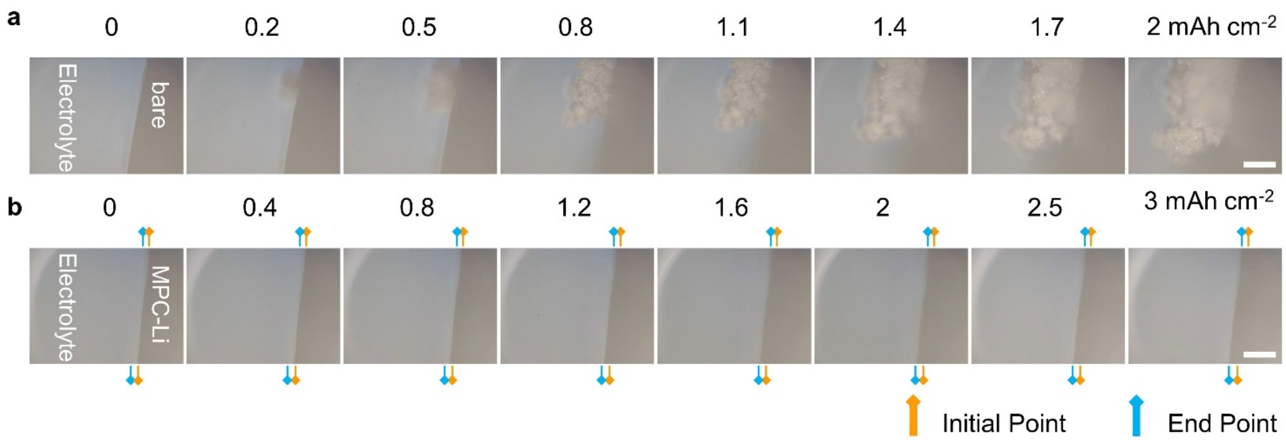

Supplementary Fig. 23 | *Operando* optical microscopy images of bare or perovskite-coated lithium metal electrodes during electrochemical deposition of lithium. a-b, the interfacial morphology change during deposition process (current density: 8 mA cm^{-2}) of the (**a**) bare Li, (**b**) MPC-Li. Scale bars: 50 µm.

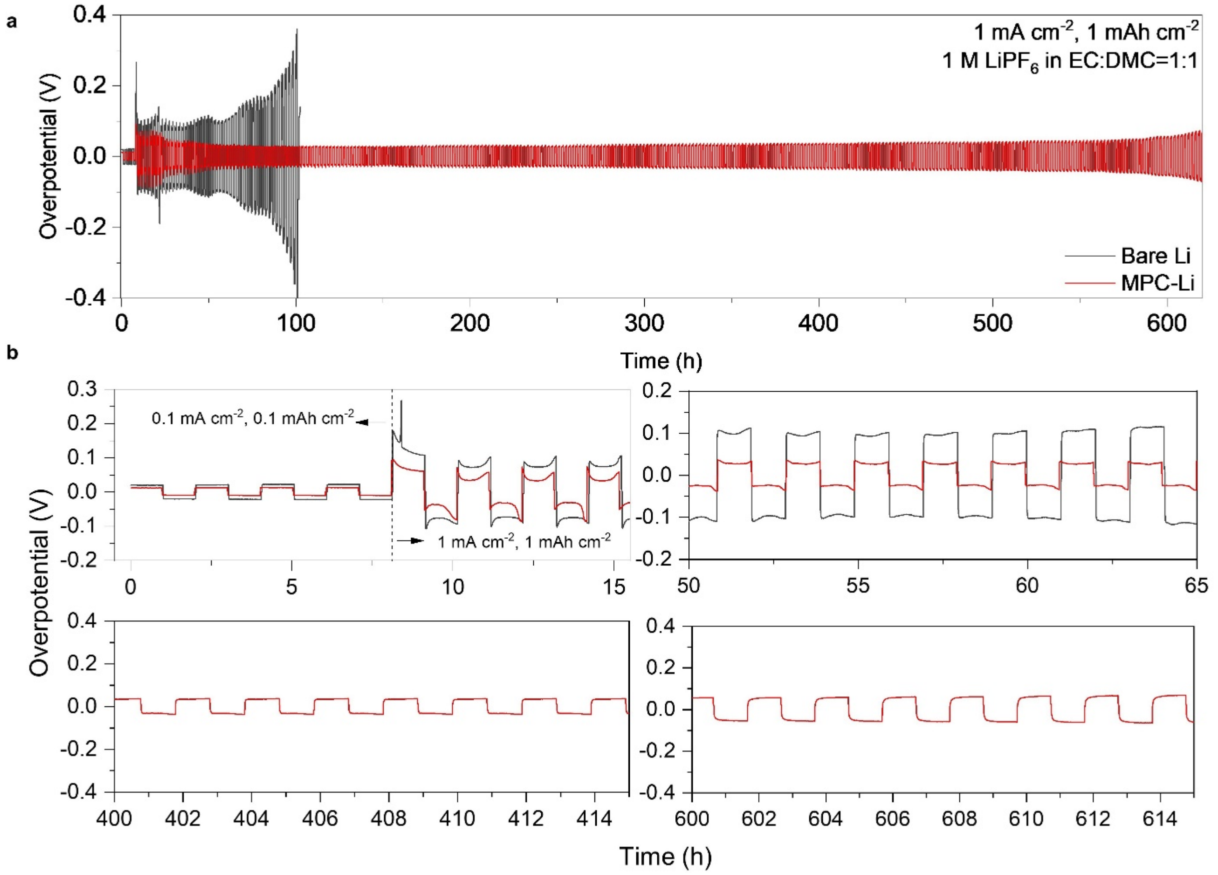

Supplementary Fig. 24 | Comparison of cycling performance of bare or perovskite-coated lithium metal electrodes in symmetric cell. a, The voltage curve of symmetric cells using bare lithium or MPC-Li protected lithium. **b,** Locally enlarged figures showing more details of the voltage curve at different periods of the cycling life.

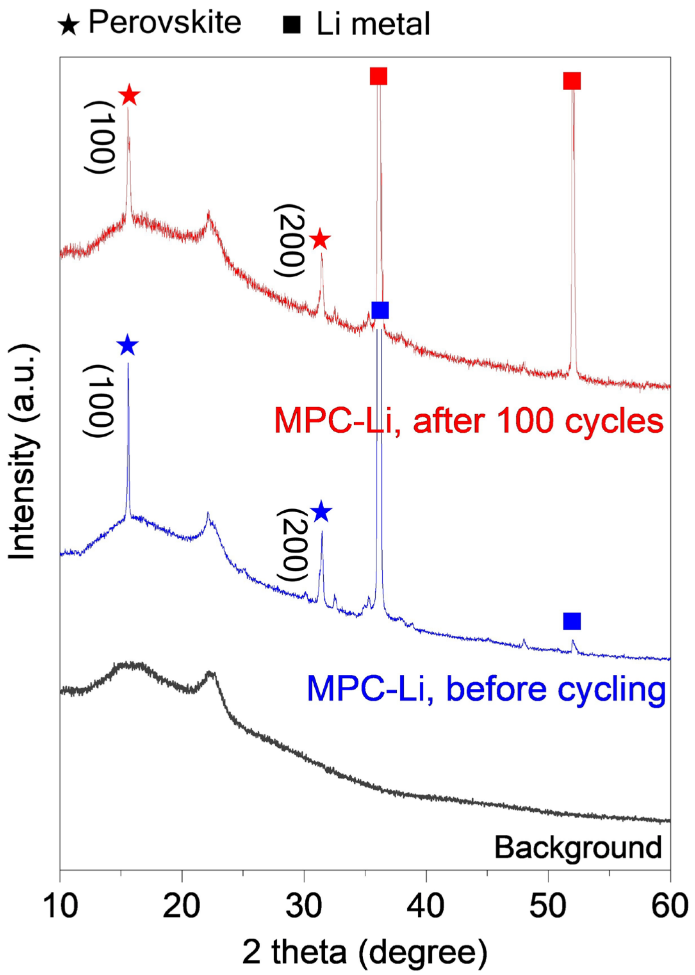

Supplementary Fig. 25 | Comparison of XRD patterns of sealing device background (black), MPC-Li anode before cycling (blue) and MPC-Li anode after 100 cycles (red). The result indicates the electrochemical stability and phase retention of the perovskite film on MPC-Li during cycling.

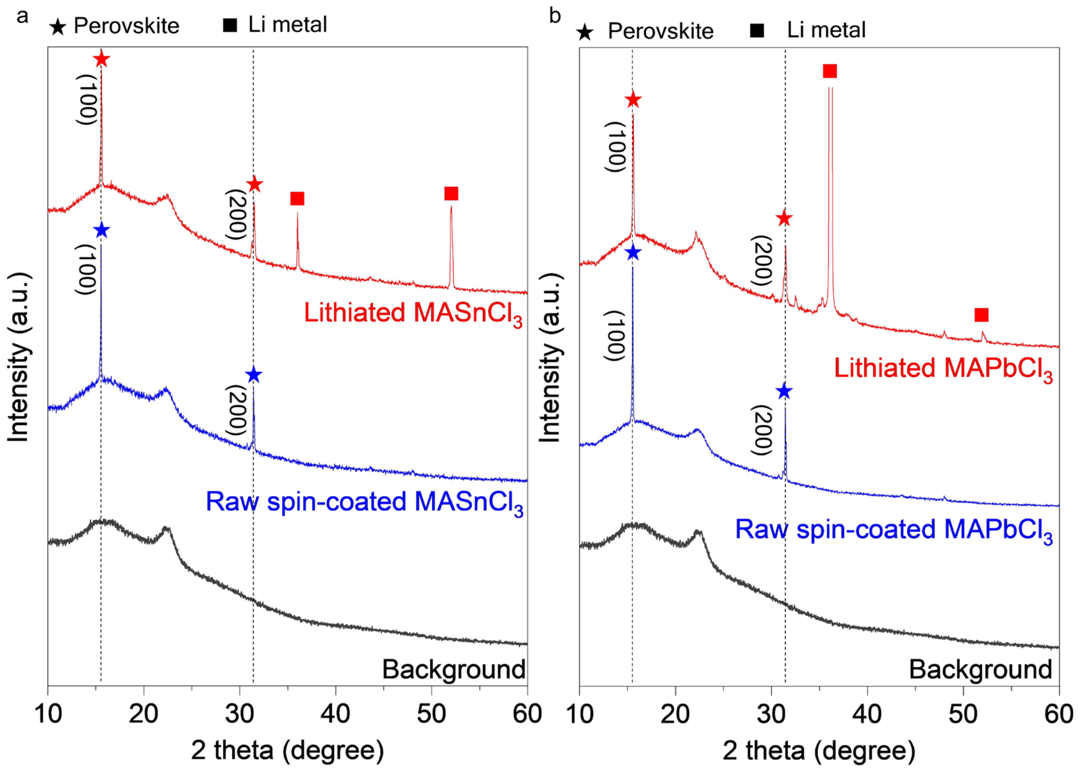

Supplementary Fig. 26 | Comparison of XRD patterns of lithiated perovskite and raw perovskite. a, XRD patterns of lithiated $MASnCl_3$ (red) and raw $MASnCl_3$ (blue), **b,** XRD patterns of lithiated $MAPbCl_3$ (red) and raw $MAPbCl_3$ (blue). The black line belongs to the XRD pattern of the sealing device.

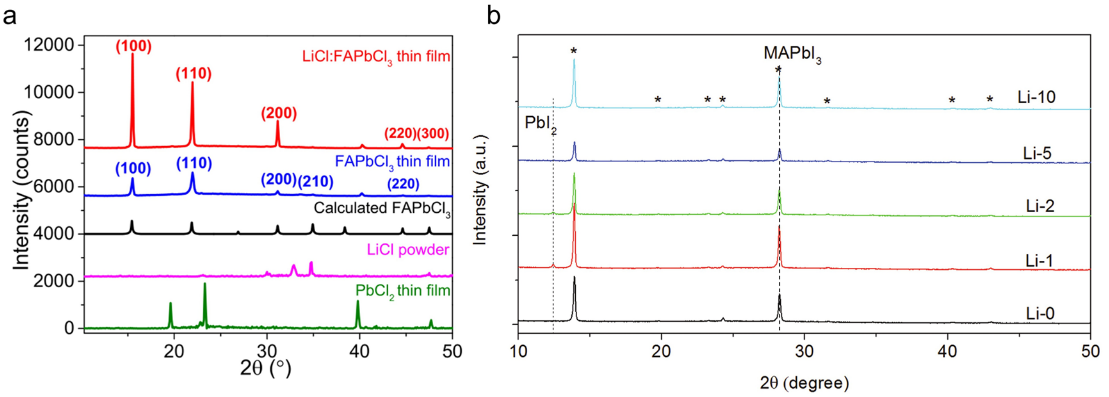

Supplementary Fig. 27 | XRD patterns of Li-doped perovskite reported in previous literatures. a, $FAPbCl_3$ nanostructures grown with LiCl additive (red, doping ratio: 10 %), without LiCl additive (blue). **b,** XRD patterns of a series of Li-doped $MAPbI_3$ films with different Li-doping ratio. (a is reproduced from Ref. 39 (main article) with permission from the Royal Society of Chemistry, and b is reproduced from Ref. 40 (main article) with permission from WILEY-VCH Verlag GmbH & Co. KGaA, Weinheim, both are cited properly in main article)

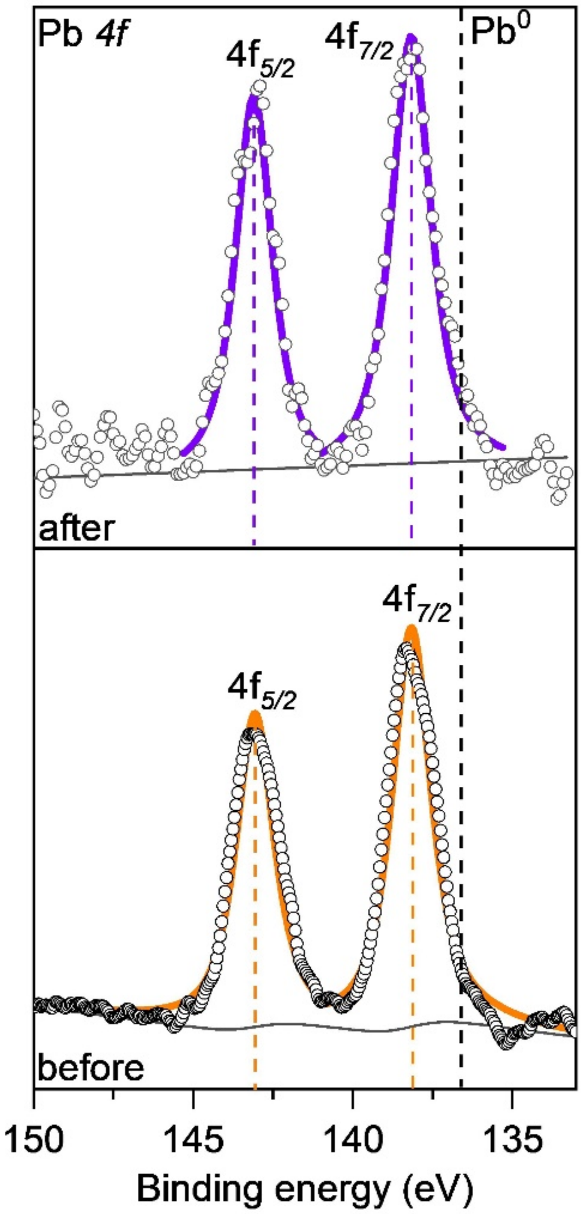

Supplementary Fig. 28 | XPS patterns of the surface of MPC-Li anodes before cycling (orange) and after 100 cycles (purple). It proves the electrochemical stability of the perovskite film on MPC-Li and that the perovskite solid-electrolyte interface stays on the top surface during cycling.

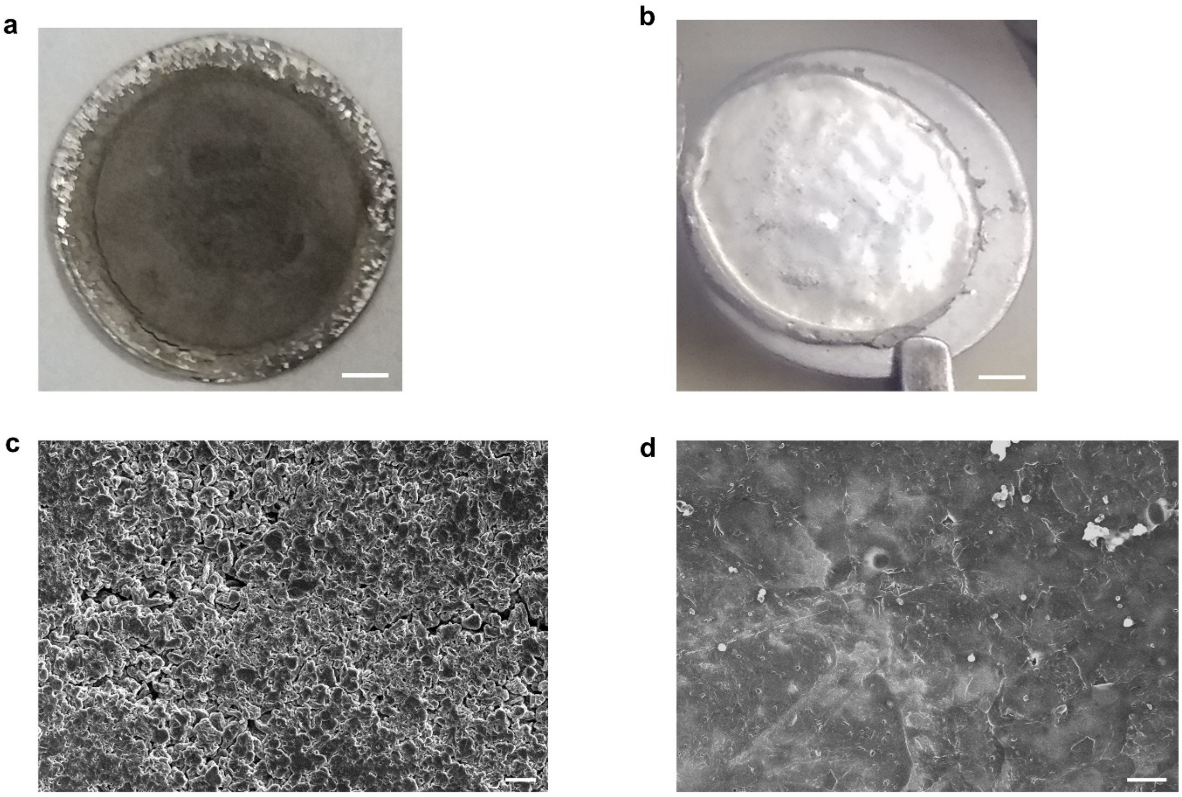

Supplementary Fig. 29 | Macroscopic photos and microscopic images of the surface of bare or perovskite-coated lithium metal electrodes after 100-hour cycling. a-b, macroscopic photos of the surface of bare lithium (**a**) and MSC-Li (**b**), **c-d,** microscopic surficial SEM images of bare lithium (**c**) and MSC-Li (**d**). Scale bars: 2 mm for (a) and (b), 10 μm for (c), and 5 μm for (d), respectively.

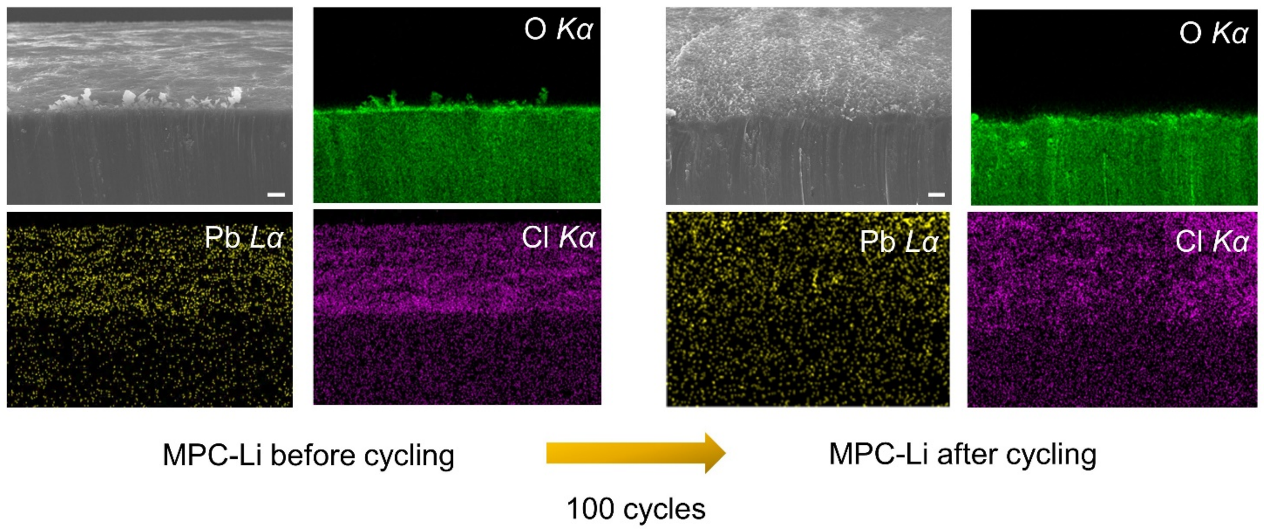

Supplementary Fig. 30 | The side-view SEM images of MPC-Li before and after 100 cycles and corresponding EDX mapping figures indicating the distribution of elements O, Pb and Cl near the surface area. The sharp contrast between the O distribution on the surface and at the side implies that MPC-Li electrode can maintain the high-quality protective layer inherited from spin-coated perovskite film during cycling.

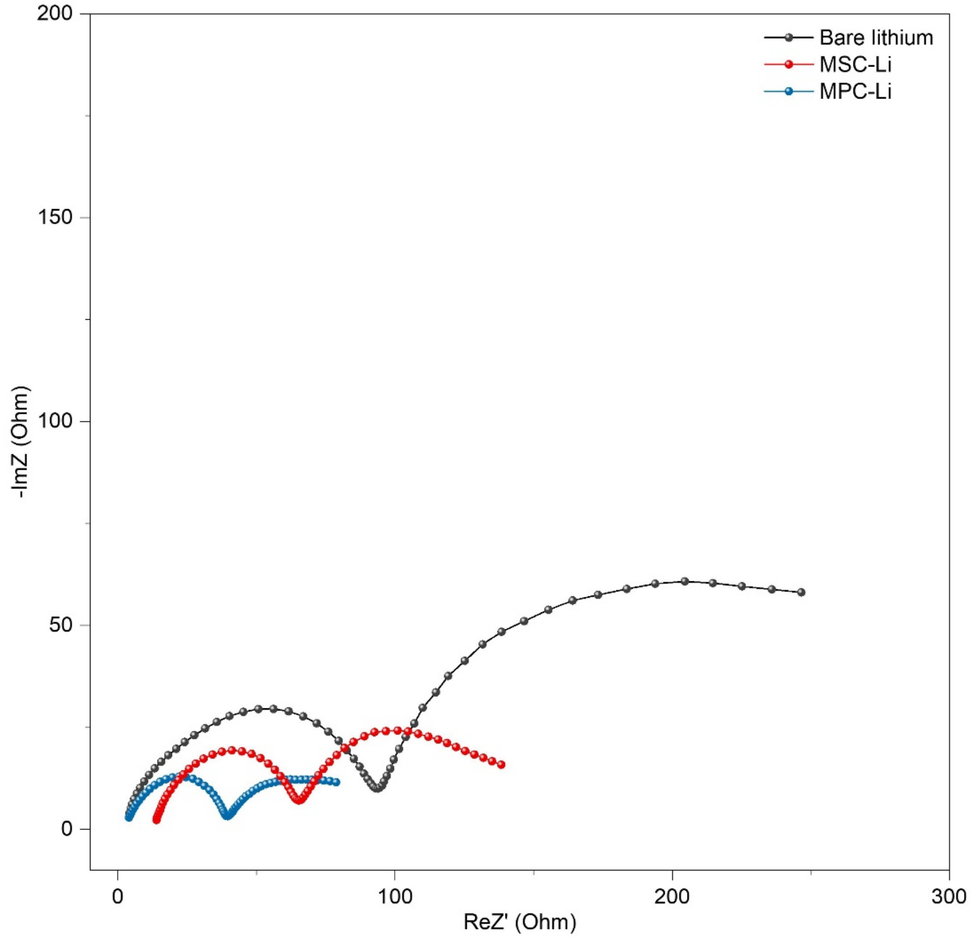

Supplementary Fig. 31 | Electrochemical impedance spectroscopies of Li | Li symmetric cells using bare lithium, MSC-Li or MPC-Li after 100 hours cycling.

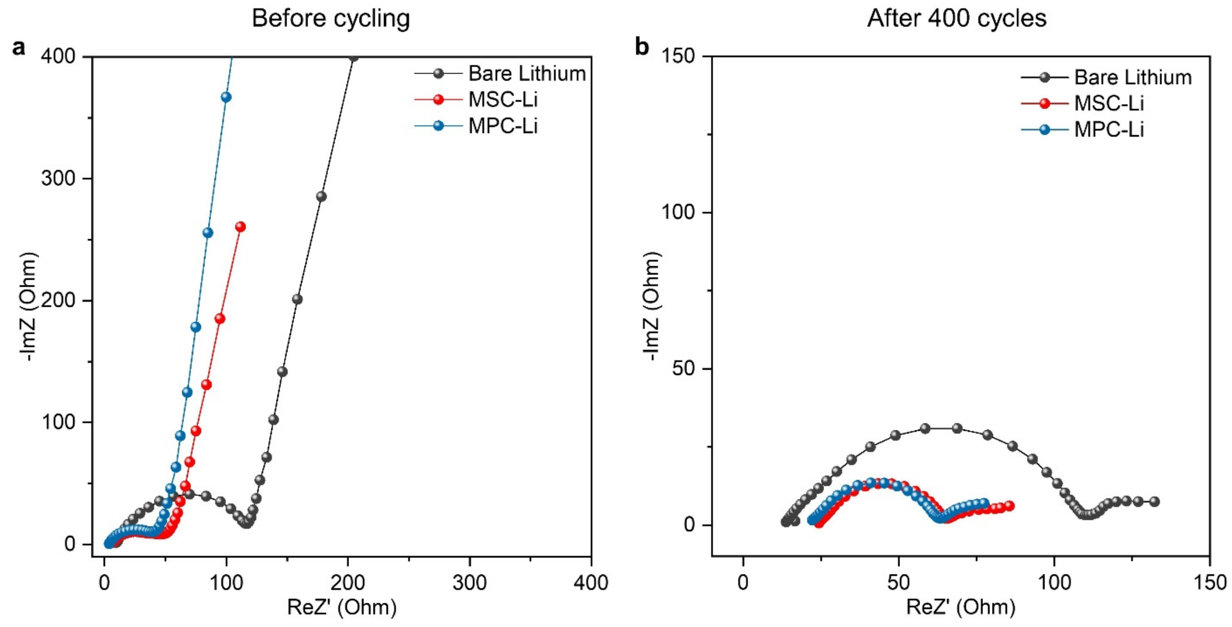

Supplementary Fig. 32 | Electrochemical impedance spectroscopies of Li | LTO cells using bare lithium metal or peorvskite-coated lithium metal as the anode. a-b, electrochemical impedance spectroscopies of Li | LTO cells using bare lithium (black), MSC-Li (red) or MPC-Li (blue) as the anode before cycling (**a**) and after 400 cycles (**b**).

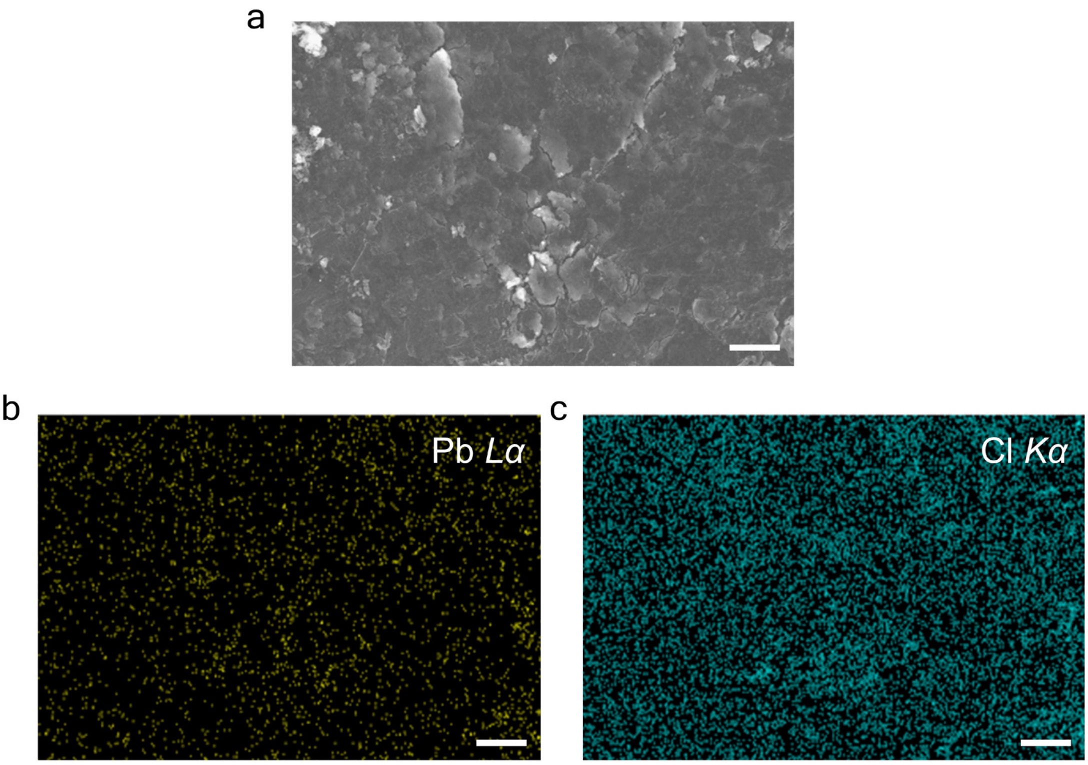

Supplementary Fig. 33 | Surface morphology of MPC-Li after being cycled for 500 cycles at 5 C and corresponding EDS mappings. a, SEM image of typical surface morphology of MPC-Li anode paired with LTO cathode after being cycled at 5 C for 500 cycles, **b-c,** corresponding EDX mappings indicating the distribution of Pb **(b)** and Cl **(c)**. The scale bars are 50 µm.

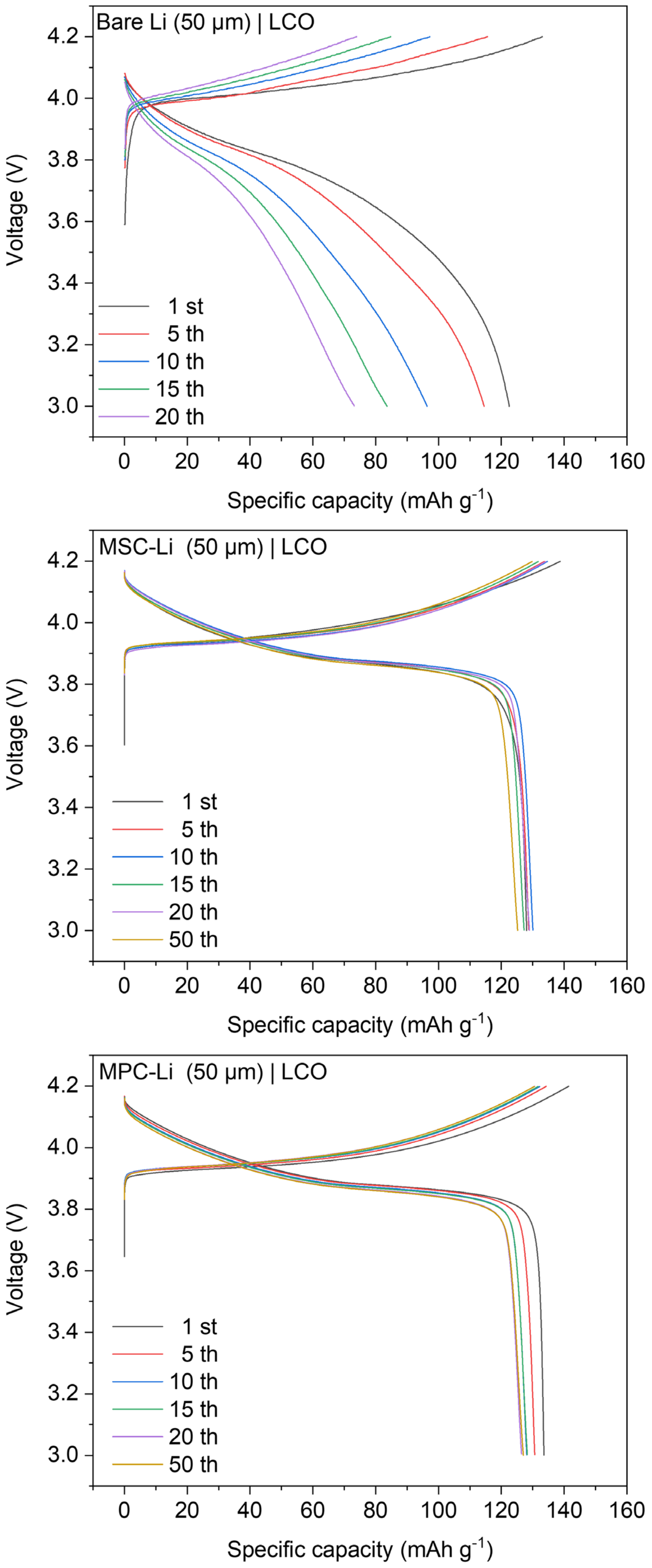

Supplementary Fig. 34 | Discharge/charge voltage profiles of Li | LCO cells at 0.5 C using bare lithium, MSC-Li or MPC-Li as the anode at different cycles. All the lithium foils used are 50 μm thick.

Supplementary Tables

Supplementary Table 1| The schematic diagram exhibiting the sampling positions (marked as white lines, 10 for each) for thickness measurement of deposited lithium on different substrates. It is the basis of the thickness statistics of Supplementary Table 2 and Fig. 1e.

	With $MASnCl_3$	With $MAPbCl_3$	Bare Substrate
2 mA cm^{-2}	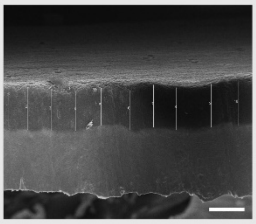	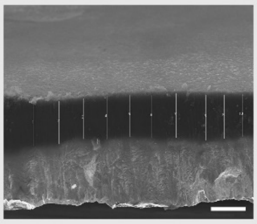	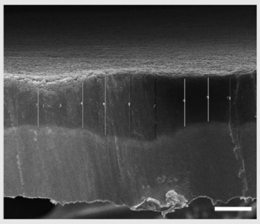
3 mA cm^{-2}	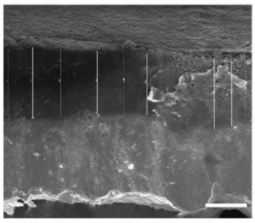	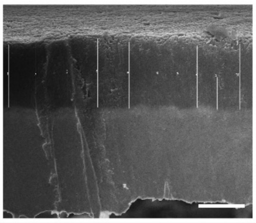	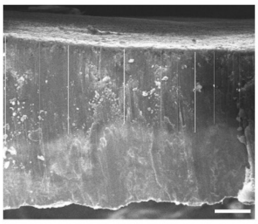
4 mA cm^{-2}	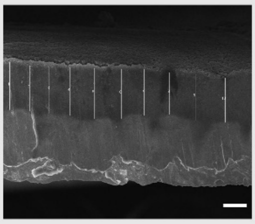	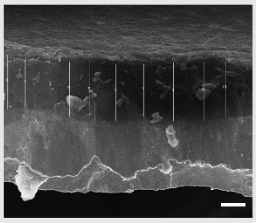	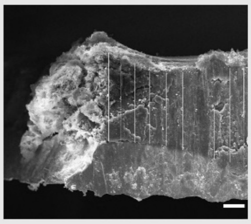
5 mA cm^{-2}	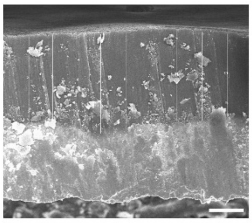	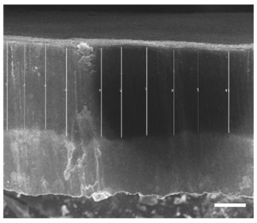	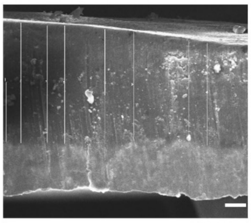
6 mA cm^{-2}	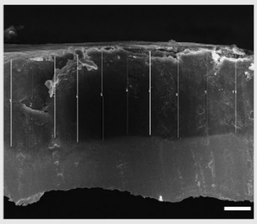	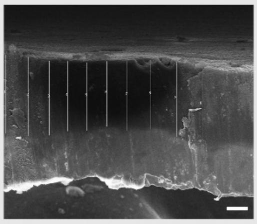	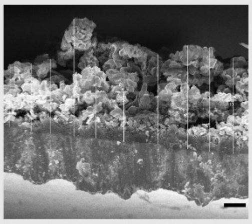

Supplementary Table 2 | The thickness measurement result, based on Supplementary Table 1, of deposited lithium on different substrates (10 sampling positions for each, unit: μm). MSC stands for deposited Li on $MASnCl_3$-coated substrates, MPC stands for deposited Li on $MAPbCl_3$-coated substrates and BS stands for deposited Li on bare substrates. This table is the data source of the box plots and error bars in Fig. 2b. For each row of the data, error bar stands for the upper and lower extremes of the range of values (UE, LE), the box stands for the 75 and 25 percentiles (UQ, LQ) and the square stands for the mean value of each set of data.

	1	2	3	4	5	6	7	8	9	10
**MSC-2**	58.05	59.32	56.77	55.93	52.96	55.50	59.74	59.74	60.59	62.28
**MSC-3**	87.22	86.12	82.47	76.27	76.64	74.81	84.66	84.66	79.55	79.19
**MSC-4**	101.27	96.79	99.35	102.55	107.68	105.12	98.07	89.09	98.07	94.22
**MSC-5**	119.99	121.73	126.51	130.42	142.60	139.55	133.90	128.25	127.38	119.55
**MSC-6**	161.22	175.26	151.67	162.91	165.72	156.17	155.60	154.48	154.48	147.18
**MPC-2**	63.47	61.73	58.69	61.73	59.56	62.17	63.91	65.65	63.47	60.86
**MPC-3**	71.83	77.02	77.02	78.74	76.67	72.87	78.39	71.15	74.26	75.63
**MPC-4**	110.36	99.38	104.26	111.57	117.06	115.84	106.70	118.89	119.50	114.01
**MPC-5**	139.41	135.08	132.20	140.85	140.37	144.22	139.41	136.53	130.28	129.31
**MPC-6**	178.75	169.16	158.20	154.78	147.93	156.83	156.15	169.16	169.16	178.75
**BS-2**	61.38	52.03	52.03	60.56	78.04	84.14	83.73	64.22	59.34	60.97
**BS-3**	150.41	142.42	122.68	134.86	103.35	106.71	113.43	111.75	99.57	107.55
**BS-4**	205.61	187.31	180.27	169.00	169.00	180.97	195.76	230.26	219.70	233.78
**BS-5**	280.96	280.96	273.92	276.03	307.01	252.79	240.12	233.78	238.71	237.30
**BS-6**	147.85	160.70	174.98	277.12	237.12	237.12	212.12	244.26	244.98	226.41

Supplementary Table 3 | The lattice parameters of Li ion contained framework of $MASnCl_3$ at different states as shown in Supplementary Fig. 3.

	$A \times B \times C$	$\alpha \times \beta \times \gamma$
**Model 00**	10.88 × 5.42 × 5.36	88.24 × 78.65 × 88.79
**Model 01**	10.91 × 5.36 × 5.41	87.59 × 75.99 × 86.90
**Model 02**	10.86 × 5.40 × 5.42	87.03 × 78.68 × 91.16
**Model 03**	10.74 × 5.40 × 5.43	87.32 × 81.72 × 95.69
**Model 04**	10.62 × 5.48 × 5.43	85.53 × 84.05 × 95.48
**Model 05**	10.41 × 5.53 × 5.41	85.19 × 83.13 × 91.72
**Model 06**	10.78 × 5.43 × 5.37	89.03 × 79.68 × 90.07

Supplementary Notes

Supplementary Note 1: (Density Functional Theory) DFT calculation of Li^+ ion transport in perovskite framework

The periodic density functional theory (DFT) calculations were performed using the Perdew Burke Ernzerhof (PBE) functional together with plane-wave projected augmented wave (PAW) method as implemented in Vienna Ab initio Simulation Package (VASP)[3]. To investigate migration of Li ions within the perovskite lattice, a $2\times1\times1$ supercell model of $MASnCl_3$ was created. The kinetic energy cutoff for the plane-wave basis set was set to be 500 eV. A $3\times6\times6$ Gamma-centered k-point grids in the first Brillouin zone were generated using the Monkhorst-Pack sampling scheme. Long-range vdW interactions have been taken into account with Grimme's DFT-D2 scheme[4]. The $MASnCl_3$ supercell was fully optimized with convergence criteria of 1×10^{-5} eV for total energy and 1×10^{-2} eV/Å for the root-mean-square residual force, respectively.

We then used the climbing-image nudged elastic band (CI-NEB) method to explore the migration path of Li ions in $MASnCl_3$, which enables us to estimate the diffusion barrier of a Li ion[5]. The convergence criteria for the CI-NEB calculation were 1×10^{-5} eV for total energy and 4×10^{-2} eV/Å for force, respectively. Since the Li ion carries a unit of positive charge, we applied the same amount of homogeneous background charge into the model system to capture its effect.

As Supplementary Fig. 2 depicts, $MASnCl_3$ possess large void space of tetrahedrons formed by Sn and Cl atoms and octahedrons by MA, Sn and Cl atoms, respectively, which makes it possible for Li ions to pass through the perovskite crystal. We manually sampled a couple of initial locations for Li using geometric and electrostatic potential information. After identifying the optimized configuration, we built the initial and final states of Li ion migration using periodic boundary conditions. We noted that the orientation of MA is correlated with Li ion as the C atom of MA always tends to be close to Li while the N atom tends to be away (Supplementary Fig. 2b and 2c). Importantly, the intercalation of Li ions into $MASnCl_3$ is thermodynamically downhill, as evidenced by the release of heat of 4.88 eV as a Li ion intercalates into $MASnCl_3$. Therefore, we continued to explore the path of Li migration in the $MASnCl_3$ crystal and associated energy barrier using DFT calculations with CI-NEB method. The simulated trajectory of Li ion migration (Supplementary Fig. 1) shows that a Li ion travels in a wiggling pattern through void space formed by Sn and Cl atoms. Three key configurations along the path are depicted in Figure 1b. We observed an appreciable distortion of the lattice and the re-orientation of MA in response to the migration of Li ions in the lattice. The energy barrier of corresponding migration paths was 0.45 eV, which is comparable to the migration barrier of Li ions in other solid-state lithium superionic conductors, like Li_4GeS_4 (0.53 eV) and γ-Li_3PS_4 (0.49 eV)[6]. Moreover, no collapse of the $MASnCl_3$ crystal has been found in the first-principle simulation of Li migration (See details in Supplementary Table 3), indicating that the crystalline framework of $MASnCl_3$ is largely maintained. This is in line with the observation of XRD and XPS results before and after cycling (Fig. 3c and 3d).

Supplementary Note 2: Reaction amount calculation of perovskite during lithiation process

In this work, the lithiation process of perovskite can be redefined as the partially reduction of the perovskite until the perovskite-coated electrode is potentially equal to Li metal in a perovskite||Li half cell (discharging from open-circuit voltage of about 2 V to the final voltage lower than 0 V). Specifically, it can be divided into two steps:

1. Intercalation of Li ions into the lattice;
2. The reduction of bottom Sn^{2+} or Pb^{2+}, and the alloying process of Sn or Pb by Li ions to form the final state alloy $Li_{17}Sn_4$ or $Li_{17}Pb_4$.

The perovskite layer is consumed only in the 2nd step. In this case, the consumption amount of perovskite can be calculated as follows:

Taking the lithiation of $MASnCl_3$ for example, the amount of perovskite consumed during lithiation is equal to the mole number of Sn lithiated by Li atoms to form the final $Li_{17}Sn_4$. The amount of Li in the alloy $Li_{17}Sn_4$ is equal to that of electrons consumed during alloy process, which starts from around 0.5 V during reduction[7,8]. The significant deviation from the balance potential is often the case with alloying process[9,10]. Thus consumption amount of $MASnCl_3$ C_{MPC} can be expressed as:

$$C_{MPC} = C_{Sn} = \frac{4}{17} C_{Li} = \frac{4}{17} C_e^{Alloy} = \frac{4}{17} C_e^{0.5\ V \to 0\ V} \tag{1}$$

The amount of electrons of 1 mAh is $3.73 \times 10^{-5}\ mol$ according to Supplementary Note 4. According to Supplementary Fig. 7, the total consumption of $MASnCl_3$ during lithiation is:

$$C_{MPC} = \frac{4}{17} C_e^{0.5\ V \to 0\ V} = 0.235 \times 0.32 \times 0.1 \quad \times 3.73 \times 10^{-0} \quad = 2.8 \times 10^{-0}\ mol\ \ cm^{-2} \tag{2}$$

For $MASnCl_3$, the mole density is[1]:

$$\rho^{MSC} = \frac{\frac{1\ cm^3}{191.1\ \mathring{A}^3}}{N^A} = \frac{10^{24}}{191.1 \times 6.022 \times 10^{23}} = 8.69 \times 10^{-0}\ \ \mathrm{mol\ \ cm^{-m}} \tag{3}$$

The quantity, from the angle of thickness, of consumed $MASnCl_3$ is:

$$d_{MSC} = \frac{C_{MPC}}{\rho^{MSC}} = \frac{2.8 \times 10^{-0}\ \ mol\ \ cm^{-2}}{8.69 \times 10^{-0}\ \ \mathrm{mol\ \ cm^{-m}}} = 3.2 \times 10^{-0} cm = 0.32\ \ \mu m \tag{4}$$

The thickness of $MASnCl_3$ film, according to Fig. 1a, is 1 µm. In this case, only about 30 % of the layer is consumed during lithiation. The lithiation process terminates when the generated LiCl is thick enough to isolate electrons coming from current collector and prevent further reduction.

The case is similar for $MAPbCl_3$, the alloying process of which starts from around 0.6 V[11-13]. As shown in Supplementary Fig. 7, the capacity belong to the process is almost the same with that of $MASnCl_3$. The thickness of $MAPbCl_3$ film, however, is twice that of $MASnCl_3$ film, so the consumption proportion of $MAPbCl_3$ film is about 15 %.

Supplementary Note 3: XPS analysis of the products including Li-M alloy and LiCl during the conversion-type electrochemical reaction.

During the conversion-type electrochemical reaction, M^{2+} at the bottom of the perovskite film is reduced and combines with Li^+ ions and electrons to form a Li-M alloy layer as shown in the following:

$$xM^{2+} + yLi^{+} + e^{-} \rightarrow Li_xM_y \tag{1}$$

We used the XPS to confirm the chemical state of the element M. Taking Li-Pb alloy layer for an example, after an electrochemical deposition of lithium (1 mAh cm^{-2}), which contains the conversion-type electrochemical reaction, the chemical state of the element Pb on the top surface of the perovskite layer was proved to be Pb^{2+} (Supplementary Fig. 9c).

To expose the bottom part of the gradient layer, where the Li-Pb alloy composition lies, the top surface part was partly removed by an adhesive tape, as shown in Supplementary Fig. 8a. In the Li-Pb alloy layer, the Pb *4f* spectrum exhibits a main peak at 136.87 eV (Supplementary Fig. 8b, yellow area), which can be assigned to Pb^0 signal for the Li-Pb alloy. Another main peak at 138.01 eV (green area) can be attributed to the residual $MAPbCl_3$ and the grey peak at 134.50 eV can be assigned to P *2p* coming from the electrolyte composition $LiPF_6$ during the initial electrochemical reaction process. Besides, the existence of alloyed Pb can be further evidenced by the signal of Li 1 s at 55.18 eV, which indicates the presence of alloyed Li (Li-Pb alloy, Supplementary Fig. 8c, green area). The side production LiCl exhibits its characteristic peak at 56.31 eV (blue area). Little amount of exposed Li metal (53.41 eV) below implies the effectiveness of the adhesive tape stripping operation to expose the bottom part of the perovskite-alloy gradient layer. Furthermore, Cl 2p spectrum in Supplementary Fig. 8d evidences the presence of $MAPbCl_3$ (200.38 eV) and LiCl (198.99 eV), which is in good agreement with the analyses of Supplementary Fig. 8b and c, indicating the formation of alloy layer under the perovskite film.

Supplementary Note 4: The theoretical thickness of lithium of unit capacity

The density of lithium[14] is 0.534 g cm^{-3}. Thus the mass of lithium of unit area (per square centimeter) is proportional to its thickness, which can be written as follows:

$$\rho = 0.534 \ \mathrm{g\ cm}^{-m} = 5.34 \ \times 10^{-0} \ \mathrm{g}\ \mu\mathrm{m}^{-g} \quad (1)$$

The molar mass of lithium is:

$$\mathrm{M} = 6.94 \ \mathrm{g\ mol}^{-o} \quad (2)$$

In this case, for a unit area, the thickness of lithium per micrometer is defined as t:

$$\mathrm{t} = \frac{\mathrm{M}}{\rho} = \frac{6.94 \ \mathrm{g\ mol}^{-o}}{5.34 \ \times 10^{-0} \ \mathrm{g}\ \mu\mathrm{m}^{-m}} = 1.30 \times 10^{5} \ \mu\mathrm{m\ mol}^{-o} \quad (3)$$

The amount of electrons consumed during deposition for a capacity of 1 mAh is:

$$\mathrm{n} = \frac{1\times10^{-\times}\times6.24\times10^{18}\times3600}{6.02\times10^{23}} = 3.73 \times 10^{-0} \ mol \quad (4)$$

Lithium ion (Li^{+}) is monovalence, which means that the amount of lithium ions reduced is equal to that of electrons consumed. Thus the theoretical thickness of lithium per square centimeter is:

$$\mathrm{T} = \mathrm{t} \times \mathrm{n} = 1.30 \times 10^{5} \times 3.73 \times 10^{-0} = 4.849 \ \mu\mathrm{m} \quad (5)$$

where T stands for the theoretical thickness per square centimeter of 100 % dense lithium under ideal consition.

Supplementary Note 5: The reactions and products of the solid-state transfer process to fabricate perovskite-lithium composite electrodes (MSC-Li and MSC-Li)

The reaction happens once the perovskite layer and the fresh lithium are pressed together as shown in Supplementary Fig. 14a. The metallic elements will be reduced to be zero valent from chloride salt.

For $MAXCl_3$, which can be seen as $MACl.XCl_2$:

$$XCl_2 + 2Li \rightarrow X + 2LiCl \quad (1)$$

where X stands for the metallic element in perovskite (e.g., Sn in $MASnCl_3$, and Pb in $MAPbCl_3$).

After the reaction, the zero valent X combines with Li to form Li-X alloy. Due to the rich quantity of lithium compared to the amount of Sn or Pb, the Li-X alloy near the Li-perovskite interface tends to be Li-rich:

$$X + 4.5Li \rightarrow Li_{17}X_4 \quad (2)$$

which is also the final state of Li-Sn or Li-Pb alloy during electrochemical lithiation. The alloy-perovskite gradient structure thus forms.

The by-product LiCl generated from the reaction is electron-isolating but ion-conducting. So it can avoid the perovskite from being reduced during electrodeposition process, ensuring the stability of perovskite during cycling, which is proved by XRD patterns before and after cycling in Fig. 3c and Supplementary Fig. 25. Furthermore, the chlorine-based perovskite $MASnCl_3$ and $MAPbCl_3$ are band-gap semi-conductor with poor conductivity of electron. In this case, the lithium deposition happens beneath instead of on the perovskite layer.

Supplementary Note 6: The spontaneous lithiation of perovskite thin film when contacted with Li metal

When applying the metal chloride perovskite thin film onto the Li metal anode, once the metal chloride perovskite thin film and Li metal were pressed together, reduction and alloying reaction occurred at the interface due to the high reduction potential of Li metal. The as-formed Li-M alloy layer mixed with LiCl at the interface is very thin and amorphous, which is beneficial for the Li^+ ion to pass through. In addition, the intercalation process of Li^+ ion into the perovskite framework is calculated (Supplementary Fig. 15a and b). This intercalation process is thermodynamically downhill, as evidenced by the release of heat of 4.88 eV as one Li^+ ion intercalates into the $MASnCl_3$ perovskite framework (Supplementary Fig. 15c), indicating a strong spontaneous trend for Li^+ ion to intercalate into the metal chloride perovskite. Therefore, as an interfacial layer on the surface of Li metal anode, the good Li^+ ionic conductivity can be retained in the metal chloride perovskite thin films, which is also confirmed by the AC impedance tests in the symmetric cells (Fig. 3a).

Supplementary Note 7: The unique gradient structure of the as-fabricated perovskite/Li-M alloy layer.

The concept "gradient thin film" refers to the gradient structure change of the protection layer from the perovskite on the top and the Li-M alloy at the bottom. Taking MSC-Li for example, the chemical state of Sn is 2+ for $MASnCl_3$ on the top and 0 for Li-Sn alloy at the bottom. In other words, the unique structure of the gradient thin film can be proved via the observation of the chemical state difference of Sn in the top layer (Sn^{2+}) and the bottom layer (Sn^0).

It is hard to distinguish the gradient structure via EDS mappings because the EDS mapping is unable to characterize the chemical state of elements. That is why the perovskite thin film seems uniformly distributed along the cross section of the film according to EDS mappings without showing the gradient structure.

To show the gradient feature of the perovskite thin film on the Li metal anode, XPS analysis was conducted. We first used Ar ion sputtering to detect the element chemical state change via the depth profiling. But the perovskite materials might be easily damaged by the Ar ion beam. So we firstly check the damage effect on pristine perovskite layers sputtered by Ar ions. For $MASnCl_3$ and $MAPbCl_3$ perovskite materials, chemical state of the B-site Pb^{2+} or Sn^{2+} ions in the perovskite would be reduced to the metal (Pb^0 or Sn^0) induced by Ar ions. As shown in Supplementary Fig. 16a, the prolongation of Ar ion sputtering time leads to more reduction of Sn^{2+} to Sn^0 (red area). The situation is similar for Ar ion sputtered $MAPbCl_3$ as shown in Supplementary Fig. 16b. Therefore, we think that the Ar ion sputtering is not suitable for depth analysis for our samples.

As an alternative way, we used adhesive tape to strip the perovskite thin film on the surface of Li metal anode for different times to check the chemical state change of the Sn or Pb (Supplementary Fig. 17a). After the first stripping, the Sn 3d spectrum consists of 2 types of characteristic peaks (top, Supplementary Fig. 17b). The yellow area exhibits a main peak at 484.19 eV (lower than the binding energy of ~484.8 eV of metallic Sn), which can be assigned to the Li-Sn alloy. Meanwhile, the blue signal area of the main peak is at 486.6 eV (Sn^{2+}), which comes from the $MASnCl_3$. The results indicate the co-existence of Li-Sn alloy and $MASnCl_3$ in the exposed layer after the first stripping. After sequent stripping for twice, the ratio of yellow area (Sn^0) to blue area (Sn^{2+}) of the main peaks increases obviously, indicating an increase of Li-Sn alloy composition and a decrease of $MASnCl_3$ part at a deeper layer. When the stripping time is up to 3, all XPS peaks can be assigned to the he Li-Sn alloy and no peaks belonging to the $MASnCl_3$ can be observed. This means that the all the Sn-contained compositions turn into the Li-Sn alloy at the bottom layer. Furthermore, $MAPbCl_3$ gradient layer exhibits the similar trend in composition change with the depth analysis implemented by the tape stripping. As shown in Supplementary Fig. 17c, the chemical state change from Pb^{2+} to Pb^0 indicates the composition transition from the $MAPbCl_3$ perovskite on the top surface to the Li-Pb alloy at the bottom layer. Together with the XPS results of the top surface of the perovskite layer as shown in the top part of Supplementary Fig. 17 b and c, the discussion and analyses above confirmed the perovskite-alloy gradient structure in the perovskite thin film on the Li metal anode.

In addition, tape stripping for 3 times is sufficient to expose the layer which directly contact with the Li metal. Taking MPC-Li for example, after stripping for 3 times, the Li 1s spectrum exhibits 3 types of characteristic peaks (Supplementary Fig. 18). The red area and the green area can be assigned to the presence of LiCl and Li-Pb alloy. The small blue area with the characteristic peak at 53.82 eV belongs to Li metal, indicating that after stripping for 3 times, the element Pb directly contacting with Li metal is exposed.

References

1. Yamada, K. *et al.* Phase transition and electric conductivity of ASnCl3 (A = Cs and CH3NH3). *Bull. Chem. Soc. Jpn.* **71**, 127-134 (1998).

2. Wan-Fu, W., Jing, S., Yong, L., Kun, Z. & Di, W. Solution Growth and Performance of CH3NH3PbCl3 Single Crystal. *J. Inorg. Mater.* **31**, 1063-1067 (2016).

3. Kresse, G. & Furthmuller, J. Efficient iterative schemes for ab initio total-energy calculations using a plane-wave basis set. *Phys. Rev. B* **54**, 11169-11186 (1996).

4. Perdew, J.P. *et al.* Restoring the density-gradient expansion for exchange in solids and surfaces. *Phys. Rev. Lett.* **100**, 136406 (2008).

5. Henkelman, G., Uberuaga, B.P. & Jonsson, H. A climbing image nudged elastic band method for finding saddle points and minimum energy paths. *J. Chem. Phys.* **113**, 9901-9904 (2000).

6. Wang, Y. *et al.* Design principles for solid-state lithium superionic conductors. *Nat. Mater.* **14**, 1026-1031 (2015).

7. Tamura, N. *et al.* Study on the anode behavior of Sn and Sn-Cu alloy thin-film electrodes. *J. Power Sources* **107**, 48-55 (2002).

8. Tamura, N., Ohshita, R., Fujimoto, M., Kamino, M. & Fujitani, S. Advanced structures in electrodeposited tin base negative electrodes for lithium secondary batteries. *J. Electrochem. Soc.* **150**, A679-A683 (2003).

9. Brousse, T., Retoux, R., Herterich, U. & Schleich, D.M. Thin-film crystalline SnO2-lithium electrodes. *J. Electrochem. Soc.* **145**, 1-4 (1998).

10. Nam, S.C. *et al.* Electrochemical characterization of various tin-based oxides as negative electrodes for rechargeable lithium batteries. *J. Power Sources* **84**, 24-31 (1999).

11. Martos, M. *et al.* Electrochemical properties of lead oxide films obtained by spray pyrolysis as negative electrodes for lithium secondary batteries. *Electrochim. Acta* **46**, 2939-2948 (2001).

12. Martos, M., Morales, J. & Sanchez, L. Lead-based systems as suitable anode materials for Li-ion batteries. *Electrochim. Acta* **48**, 615-621 (2003).

13. Ng, S.H. *et al.* Spray pyrolyzed PbO-carbon nanocomposites as anode for lithium-ion batteries. *J. Electrochem. Soc.* **153**, A787-A793 (2006).

14. Lide, D. R. *CRC Handbook of Chemistry and Physics, 89th Edition (Internet Version 2009)*, Ch. 4, 20 (CRC Press/Taylor and Francis, Boca Raton, FL., London, 2009).
